# Vulnerabilities of Arab refugees in primary health care: a scoping review

**DOI:** 10.11606/s1518-8787.2022056003691

**Published:** 2022-03-23

**Authors:** Luiz Paulo de Lima, Kayte Chaves Oliveira de Lima, Maria Rita Bertolozzi, Francisco Oscar de Siqueira França

**Affiliations:** I Universidade de São Paulo Faculdade de Saúde Pública Programa de Pós-Graduação em Saúde Global e Sustentabilidade São Paulo SP Brasil Universidade de São Paulo . Faculdade de Saúde Pública . Programa de Pós-Graduação em Saúde Global e Sustentabilidade . São Paulo , SP , Brasil; II Universidade de São Paulo Escola de Enfermagem Programa de Pós-Graduação em Enfermagem São Paulo SP Brasil Universidade de São Paulo . Escola de Enfermagem . Programa de Pós-Graduação em Enfermagem . São Paulo , SP , Brasil; III Universidade de São Paulo Escola de Enfermagem Departamento de Enfermagem em Saúde Coletiva São Paulo SP Brasil Universidade de São Paulo . Escola de Enfermagem . Departamento de Enfermagem em Saúde Coletiva . São Paulo , SP , Brasil; IV Universidade de São Paulo Faculdade de Medicina Departamento de Moléstias Infecciosas e Parasitárias São Paulo SP Brasil Universidade de São Paulo . Faculdade de Medicina . Departamento de Moléstias Infecciosas e Parasitárias . Núcleo de Medicina Tropical. São Paulo , SP , Brasil

**Keywords:** Refugees, Arabs, Health Services Needs and Demand, Primary Health Care, Health Vulnerability, Review

## Abstract

**OBJECTIVE:**

To map and analyze the vulnerabilities of Arab refugees in the context of primary health care.

**METHOD:**

Scoping review in which studies published in English, Spanish and Portuguese languages from 2011 onwards were reviewed. The following databases were surveyed: Cochrane, Scopus, Health System Evidence, MedLine-PubMed, CINAHL, Embase, Lilacs, Web of Science, SciELO, NYAM Grey Literature, BVS, Capes Thesis and Dissertation Database, Refworld and Journal of Refugee Studies. Data were analyzed in light of the concept of vulnerability.

**RESULTS:**

Of the 854 studies identified, 40 articles were held for analysis and extraction of vulnerability indicators in the individual, social and programmatic dimensions. Regarding the individual dimension, the main indicators identified were unemployment, unstable and overcrowded housing, lack of sanitation and access to water, mental disorders, communicable and chronic noncommunicable diseases, etc. In the programmatic dimension, were identified, mainly, health teams with work overload, lack of preparation to deal with cultural and linguistic barriers, and delays in providing care. In relation to the social dimension, lack of access to schools, to information about health programs in the host countries, and to rights, among others, were found.

**CONCLUSION:**

Vulnerabilities found highlight the disadvantage of refugees regarding health programs, services and system in host countries, in addition to highlighting the deep inequalities that affect this group. It is pointed out the need for programs and policies that promote actions, within the scope of primary health care, which recognize and respond to the health needs of refugees.

## INTRODUCTION

In 2019 it was estimated that 275 million people migrated outside the borders of their own countries, meaning that 3.5% of the world’s population were international migrants. That figure number has tripled in the last 45 years ^1^ . Currently, there are about 79.5 million people in forced displacement and, of these, 26 million are refugees ^2^ , i.e., people who “are out of their home country because of well-founded fear of persecution related to armed conflict, race, religion, nationality, membership of a particular social group or political opinion, or serious and widespread violation of human rights” ^3^ .

**Figure:**
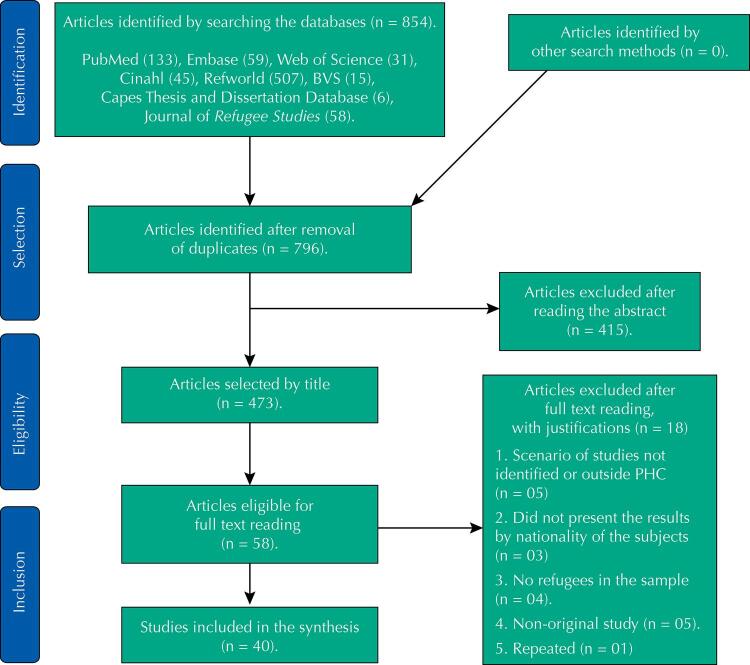
Flow diagram on the process of selecting studies for Scope Reviews, inspired by PRISMA by Moher et al. 15 .

In 2011, due to the so-called Arab Spring and the armed conflict in Syria, the Arab world became the epicenter of the refugee issue. Syria now has the largest forcibly displaced population in the world. Of its population of 13.5 million in 2019, 6.7 million of them are refugees. Iraq has about 3.3 million people in this situation, and Palestine, 5.5 million ^2^ . Lebanon has the highest concentration of refugees in the world in relation to the general population (156/1,000) ^4^ , which worsens health conditions in the country, noting that most refugees come from Syria ^5^ .

Global refugee trends also echo in Brazil. Since the beginning of the conflict in Syria, 3,772 people have requested refuge in the country ^9^ and, among the Arabs, Syria, Palestine, Lebanon and Iraq are the countries of origin that had their refugee status most often recognized in Brazil ^10^ .

The status of refugee is *per se* an element of vulnerability considering forced migration, and because it represents a serious and widespread violation of human rights ^11^ . This article aims at mapping and reviewing the elements of health vulnerability of Arab refugees in the scope of primary health care (PHC).

## METHOD

This is a 5-stage scoping review, following Arksey and O’Malley ^12^: 

### Stage I: Identification of the research question.

What are the elements of vulnerability, in the individual, programmatic, and social dimensions, that impact Arab refugees in the context of primary health care?

### Stage II: Identification of relevant studies.

Two strategies were used ( Table 1 ), adapted according to the specificity of each database. Following databases were visited: Lilacs, SciELO, NYAM Grey Literature, Cochrane, Health System Evidence, MedLine/PubMed, Embase, Web of Science, CINAHL, BVS, Capes Theses and Dissertations Database, Refworld, and Journal of Refugee Studies. Studies published from January 1, 2011 to December 31, 2019 were included, considering the historical context of the Arab Spring.

**Table 1:** Strategies of descriptors-based (MeSH) and keywords-based search:

**Descriptors and keywords for the search strategy 1** “Primary Health Care” [Keyword, MeSH]; OR “Primary Care” [Keyword]; OR “General Practice” [Keyword, MeSH]; OR “FamilyPractice” [Keyword, MeSH]; AND Refugee* [Keyword, MeSH]; OR “Asylum Seeker” [Keyword]; AND Arab [Keyword]; OR Syria [MeSH, Keyword]; OR Syrian [Keyword]; OR Palestinian [Keyword]; OR Palestine [Keyword]; OR Iraqi [Keyword]; OR Iraq [Keyword, MeSH]; OR Lebanese [Keyword]; OR Lebanon [Keyword, MeSH].
**Descriptors and keywords for the search strategy 2** Vulnerability [Keyword]; AND Arab [Keyword]; OR Syria [MeSH, Keyword]; OR Syrian [Keyword]; OR Palestinian [Keyword]; OR Palestine [Keyword]; OR Iraqi [Keyword]; OR Iraq [MeSH, Keyword]; OR Lebanese [Keyword]; ORLebanon [Keyword, MeSH].

### Stage III: Study selection.

Following were the inclusion criteria: subjects of the studies were Arab, Syrian, Palestinian, Iraqi, and Lebanese refugees in the condition resulting from forced migration, because they are the nationalities that most had the refugee status recognized in Brazil ^10^ ; studies that made it possible to identify constitutive elements of the concept of vulnerability; studies that presented elements of vulnerability in any of the four phases of refuge (origin, transit, destination, and return); studies that had PHC as a setting.

The following studies were excluded: on voluntary migration (non-forced displacement and economic migration); did not bring original/primary data (article of opinion, experience reports, literature reviews). We did not delimit the length of migration of the studies’ subjects.

We consider vulnerability “a set of individual and collective aspects related to the greater susceptibility of individuals and communities to an illness or injury and, inseparably, the lower availability of resources for their protection” ^13^ . We also consider the three interdependent, inextricable, and synergistic dimensions of the concept: individual, programmatic, and social ^13^ . The individual dimension takes as its starting point aspects of the way of living that may contribute to the exposure to a given health problem, or aspects that may protect against certain problems. The programmatic dimension integrates the efforts of institutional programs, especially health programs. The social dimension covers all the contextual elements related to life in society: the legal, political, and ideological structure, governmental guidelines related to health and social issues, social relations, and religious beliefs, among others ^14^ .

After identifying the articles in databases, duplicates were excluded, followed by analysis by title; by abstract; and by the full text ( Figure ).

### Stage IV: Mapping the studies.

We designed a form to extract and organize the following data: author, year of publication, country of study, country of origin and destination of refugees, objectives, type and population of the study, sample size, study setting, elements of vulnerability in the individual, programmatic and social dimensions, as indicators of stage V (Tables 2, 3 and 4).

### Stage V: Conference, summary, and reporting of results.

Data were analyzed and summarized according to the following vulnerability indicators proposed by Nichiata, Takahashi and Bertolozzi ^16^: 

Individual dimension: 1) Demographic profile (gender, age, occupation); 2) Family structure (nuclear, non-nucleated, number of children); 3) Living conditions (domicile: tents/containers/urban house, basic sanitation); 4) Work (profession, occupation, working hours); 5) Social relations at work (owner, employee); 6) Morbidity and mortality profile; 7) Beliefs and values about the health-disease process.

Programmatic dimension: 1) Health Policies; 2) Actions of the Health Programs; 3) Access to Health Services.

Social Dimension: 1) Access to: education, culture, information, leisure and justice; 2) Participation in collective actions; 3) Religious belief; 4) Prejudice/stigma; 4) Migration (origin, transit, destination and return).

## RESULTS

After selection, 40 studies remained for analysis and data extraction ( Figure ). In the process of reviewing the production of studies by continent it was found that Asia produced 15 studies (37.5%), North America 12 (30%), Europe 12 (30%), and Oceania 1 (2.5%). No original study published in a Brazilian or Latin American journal was found. No relevant grey literature was identified either. Studies on Syrians were prevalent (18: 45%), followed by Palestinians (13: 32%) and Iraqis (2: 5%). The predominance of studies with Syrians is justified by the historical interval studied (from 2011 onwards). We did not identify studies with Lebanese refugees. Most of the studies (30) were conducted with refugees in the destination countries: Jordan (17 studies: 42%); Lebanon (9: 22%); Syria (3: 7.5%); in urban areas (29: 72%). Most of the studies (31: 77%) were quantitative.

*Health Vulnerabilities of Refugees*: *Categorization by dimensions (individual, programmatic and social) and country of origin*

### I - Syrian Refugees

Twenty-two studies were found ( Table 2 ) ^17^ . Regarding the elements of individual vulnerability, the morbidities that stood out most were: mental disorders, overweight/obesity, eating disorders, tuberculosis, chronic noncommunicable diseases, and sexually transmitted infections. Syrian refugees are about 10 times more susceptible than the host country population to mental health problems (52%) ^32^ and post-traumatic stress disorder (PTSD) was present in almost half of the cases ^28^ , strongly associated with exposure to war ^32^ and eating disorders ^28^ . Anxiety and depression were also highly prevalent (40.3% and 47.7%, respectively). Most of the refugees who developed anxiety and depression in the country of destination did not have it in the country of origin ^36^ . The conditions that may result from adverse experiences at the destination (exposure to trauma, loss of family members, in relation to social status and material goods, chronic deprivation in basic needs); in the transition (unsafe means of transportation: risk at sea crossing, of exploitation by smugglers), in the camps (overcrowding, uncertainty about the future, and severe lack of resources), and after resettlement (unfamiliarity with the new context, language barriers, poverty, unemployment, lack of support, discrimination) ^36^ .

**Table 2:** Health vulnerabilities of Syrian refugees.

Author/year	Type of study	Objective of the study	Population of the study	Vulnerabilities identified		

				Individual	Programmatic	Social
UNHCR/UNICEF ^17^ (2016)	Qualitative and quantitative mixed method semi-structured interview, survey and focal group.	Provide multi-sector view on the status of Syrian refugees in Lebanon.	23,000 Syrian refugees, 4,561 children younger than 5 years.			
				93%: some degree of food insecurity (1.8 meals/day). Chronic (43%) and mental (12%) diseases. Children: 41% sick in the two weeks prior to the study. Symptoms: fever (31%), cough (25%) and diarrhea (15%). Overcrowded housing and no toilets (27%).		84% of children of 15 to 17 years old out of school. Causes: cost of education, child labor and marriage, cultural reasons, and cost of transportation. 70%: below poverty line ($3.84/day/person).
Hosten E. et al. ^18^ (2018)	Quantitative Retrospective Study.	Assess the prevalence of active and latent Tuberculosis (TB), risk factors for latent TB, and the performance of the Jordanian TB program.	76 Syrian refugees with TB (index cases). 481 contacts.	High prevalence of active and latent TB among contacts of TB patient. In contacts: prevalence of active and latent TB in children under 5 years old is 2 times higher than among adults.	Cure rate among index cases: 28.9%. Among women: 7.9%. Low adherence to treatment: 43.4%.	
Truppa. C. et al. ^19^ (2019)	Quantitative Cross-sectional Survey.	Determine the impact of primary health care (PHC) on vulnerable populations. Identify barriers to the use of health services.	656 Syrian refugees in Lebanon.	Most common reasons for seeking care: Chronic noncommunicable diseases (40.6%): arthritis, musculoskeletal conditions, hypertension and diabetes; sexual and reproductive health conditions (28.6%). Communicable diseases affecting children (37.8%).	Most important barrier to using the services: lack of access to information about the services offered (54.2%). Low vaccination coverage. Low level of prenatal care and family planning. 80.9% had to pay additional amounts for health services in PHC.	-
Doocy. S; Lyles, E. ^20^ (2017)	Quantitative Unidentified Survey Design.	Identify unmet needs and priorities for assistance in families undergoing displacement, and headed by women.	2,045 Syrian refugee families.	Families undergoing displacement and headed by women: most vulnerable. Food insecurity.		
Ay, M. et al. ^21^ (2016)	Quantitative Cross-sectional Analytical Observational.	Identify health care service needs, accessibility to services, and barriers to access	196 Syrian refugees in Jordan.	56.6% sought PHC for acute illness in adults, and 53% in children. 36% reported chronic illnesses.	Discrimination by staff. Barriers to access: cost, long waiting hours, distance to health service, late appointments.	Lack of information about the provision of free of charge family planning services.
Al-Rousan, T. et al. ^22^ (2018)	Qualitative-quantitative. Mixed Methods.	Determine health needs from the perspective of refugees, health care team, and other stakeholders.	185 Syrian refugees in Jordan. 75 in the camp (41 men) 110 urban (65 women)	Respiratory illnesses associated with the desert climate of the camp. Chronic illnesses.	Shortage of transportation to reach the UBS. Shortage of female medical professionals for perinatal care. Overloaded health team.	Due to stigma, they don not report mental health care needs Poor housing conditions.
Cherri, Z. et al. ^23^ (2017)	Qualitative Focal Group Interview thematic analysis.	Understanding needs, preferences, behaviors, and barriers to reproductive and sexual health.	108 Syrian refugee women (15 to 49 years old) in Lebanon.	High birth rate worsening socioeconomic condition. Early marriage (14 years) due to economic scarcity.	Cost reported as barrier to access. Lack of access to information on contraceptive methods.	42% did not use any contraceptive method. Forced migration reported as cause for non-use.
Doocy, S. et al. ^24^ (2015)	Quantitative Cross-Sectional Study.	Characterize the prevalence of Noncommunicable Diseases.	9,580 Syrian refugees in Jordan (1,550 families) - 0 to over 60 years old	50% report a family member with a chronic noncommunicable disease.	Health policies that abrogate rights (in 2014: established payment of fees to obtain public health care).	Low access to education for heads of household. Lack of access to information regarding the supply of health services.
Gammoh, O.S. ^25^ (2016)	Quantitative Cross-Sectional Study.	Describe complaints, diagnoses, and medication consumption.	375 Syrian refugees in Jordan, 0 to over 70 years old, 213 Women (56.8%). 162 Men (43.2%).	Infectious, chronic, cardiovascular (hypertension), musculoskeletal, gastrointestinal, respiratory, and skin diseases. Complaints: pain (throat, gastrointestinal), cough, shortness of breath, skin rash, fever, dizziness, weakness, anxiety symptoms, dental and vision problems.		
Kakalou, E. et al. ^26^ (2018)	Descriptive Quantitative Cross-Sectional Study.	Describe the morbidity profile of the refugee population in Europe in 2015-2016.	3,076 Syrians, 1,270 Iraqis.	Infectious diseases. Musculoskeletal, skin conditions, trauma/injury (caused by accident/violence). Cardiovascular, endocrine, respiratory, neurological, autoimmune, and congenital diseases Dental or oral cavity conditions. Women’s reproductive conditions, mental health, malnutrition, weakness, anemia, scabies and lice, substance abuse (psychotropic and alcohol).	50% of consultations with specialists were offered by NGOs (failure of the local health system to offer expert personnel).	
Sethi. S. et al. ^27^ (2017)	Quantitative Unidentified Study Design.	Identify care gaps in noncommunicable diseases.	640 Syrians in Lebanon. 320 adults over 18 years of age. 300 children under 2 years of age.	Communicable and noncommunicable diseases.	Lack of transportation, lack of time to go to health facility and cost: reported as barrier in access.	Low education of mothers: 40.3% have no formal education. 20% with complete primary education
Aoun, A; Joundi, J; El Gerges, N. ^28^ (2018)	Quantitative Cross-Sectional Study.	Examine the prevalence of eating disorders, and association with sociodemographic and clinical variables.	450 Syrians in Lebanon. 69 Men and 381 Women (18 to 45 years old).	Post-traumatic stress disorder, eating disorders, overweight and unemployment.	-	48% can only read and write in an elementary way.
Collins, D.R.J. ^29^ (2017)	Qualitative-Quantitative Mixed Methods.	Determine the cardiovascular disease risk profile of the population.	2,907 Syrians in Jordan (18 to > 40 years old). 16 PHC team members.	Cardiovascular disease, dyslipidemia, diabetes and sedentary lifestyle.	Limited knowledge of health care team regarding protocols for cardiovascular disease.	Migration pattern impacted adherence to drug treatment.
Dogru, S; Doner, P. ^30^ (2017)	Quantitative Retrospective and Descriptive.	Compare the frequency of new cases of pulmonary tuberculosis, and successful treatment.	33 Syrian refugees with Tuberculosis in Turkey.	Tuberculosis affects younger age groups among Syrians compared to the local population.	TB treatment goals were not met Lower cure rate among Syrians.	
Doocy. S. et al. ^31^ (2016)	Quantitative Cross-sectional	Assess health status, unmet needs, and health service delivery.	1,376 families/8,257 Syrians in Lebanon (0 to > 60 years old).	Hypertension, cardiovascular disease, diabetes, chronic respiratory disease, and arthritis.	Need to pay for health services in PHC.	
Segal, S. P. et al. ^32^ (2018)	Quantitative Cross-sectional Structured Interview Survey.	Assess the mental health status of Syrian and Palestinian refugees. Determine the prevalence of mental illness.	161 Palestinians, 47 Syrians and 46 Lebanese in Shatila camp in Lebanon.	High prevalence (52%) of mental disorders (10 times higher than local population).	Need to strengthen the mental health program in PHC.	High rate of people without schooling: up to 3 times more than the local population. Difficulties in access to justice, laws that restrict fundamental rights. Low access to the local economy. Live an average of 17 years in the camps. Lack of basic sanitation. Overcrowded camps. Insufficient housing.
Heenan, R.C. et al. ^33^ (2019)	Quantitative Retrospective Study.	Review health conditions of Syrian and Iraqi refugee children in the context of screening at arrival in Australia.	128 children (7 months to 16 years).	Vitamin D deficit (63%), growth/nutrition deficits, neurological/metabolic disorders, related to learning/behavioral mental health, latent tuberculosis, developmental delay.	The most omitted screening tests were for tuberculosis (only 7.1% completed screening). Delayed vaccination schedule in children. Difficulty in access to tuberculin skin testing/PPD in PHC.	25% of children with difficulty in accessing education even after 3 months in the host country. Pre-arrival adversities: exposure to trauma, concern about parents’ mental health, concern about other families abroad, separation from a family member
Doocy, S. et al. ^34^ (2017)	Qualitative-Quantitative Mixed Method Longitudinal Cohort Study.	Evaluate the effectiveness of treatment guidelines and a mobile health app (mHealth) on quality of care and health outcomes	793 Syrian Refugees in Lebanon with Type 2 Diabetes and Hypertension.	Uncontrolled diabetes (57.9%).	Team having difficulty in interacting with patient. Low percentage of patients who received lifestyle health counseling. Divergences between electronic data and written medical records.	
Elliot. J.A. et al. ^35^ (2018)	Quantitative Cross-Sectional Study.	Determine associations between patient factors, self-management, diabetes education and support.	292 Syrian Refugees in Lebanon (>18 to 84 years old) with Diabetes.	Diabetes (diagnosed in 30% of patients after the conflict in Syria). Long fasting (Ramadan) associated with insulin use.		Low educational level. Inability to recognize and respond to hypoglycemia (34%).
Javanbakht. A. et al. ^36^ (2018)	Quantitative Cross-Sectional Study.	Determine the prevalence of mental disorders.	157 Syrians in the United States (18 to 65 years old).	Post-traumatic Stress Disorder (PTSD) (32.2%), anxiety (40.3%), and depression (47.7%), mainly after forced migration.		
Torun. P. et al. ^37^ (2018)	Qualitative-Quantitative Mixed Method.	Assess health needs of urban refugees in Istanbul.	111 families/8,901 Syrian refugees in Turkey.	Symptomatology compatible with sexually transmitted infection, chronic, pulmonary, cardiac, gastrointestinal, neurological, renal, psychiatric, musculoskeletal diseases, hypertension.	Pregnant women (60%) with difficulties in access due to language barrier, waiting time, queues, tense environments, and negative attitudes of the staff.	High rate of children dropping out of school due to migration (55%). Lack of aid for education and rent. 49.6% did not know about the right to free access to health care. Inadequate working conditions.
Lyles E. et al. ^38^ (2018)	Quantitative Unidentified Design Survey.	Characterize access to and utilization of health services in Lebanon.	2,062 Syrian refugee families in Lebanon.	Communicable, and chronic noncommunicable diseases, injuries, skin diseases, pregnancy complications, gynecological, gastrointestinal, and eye problems.	Greater difficulty in access to medicines than local population.	

Chronic noncommunicable diseases, especially diabetes (DM) and hypertension (SAH) were mentioned in eight studies ^22 , 24 , 27 , 31 , 34 , 35 , 38 , 54^ , and the prevalence of DM was higher than in the local population; in women, it was almost twice as high, as was the risk for the disease ^54^ . The higher prevalence of DM refers mainly to individuals with a relatively long-lasting history of migration. There is an increased risk for DM about six months after arrival, and it is accentuated after four years of migration ^54^ . Refugees may face an accumulation of risk factors for DM: increased genetic susceptibility, low birth weight, exposure to childhood hunger, major socioeconomic change, acculturation stress, and distinct lifestyles in the host country. The refuge seekers with PTSD had almost 1.5 times greater chance of being diagnosed with DM compared to others in the same condition ^54^ . Also, fasting blood glucose levels above 120 mg/dl were found in 57.9% of the refugees with DM ^34^ .

Approximately 73.9% of the refugees had a family member with a chronic disease (SAH: 25.2% of patients) ^37^ . Respiratory diseases were mentioned in four studies ^22 , 25 , 27 , 31^ . In Lebanon, 89.1% of Syrian refugees in the study sample sought PHC due to chronic respiratory disease ^31^ . Refugees experience exacerbation of respiratory diseases due to the desert climate of some refugee camps ^22^ . Tuberculosis (TB) was noted in three studies ^27 , 30 , 33^ . About 11.8% of Syrian children (aged 7 months to 16 years) in Australia had tuberculosis ^33^ . Tuberculosis treatment success was lower among Syrian refugees in Turkey (63.6% of cases) than among local population (88.8%). The context of war is directly related to the increased prevalence of the disease. In Syria, before the beginning of the conflict, the incidence was 23 cases/100,000 inhabitants, and after the conflict it increased to 51 cases/100,000 ^30^ . During the civil war, the main problems in relation to tuberculosis are related to diagnosis, treatment, and prevention of the disease, access to health services and drug supply, increased reactivation, transmission, due to living conditions: crowding, nutrition, shortage of medicines and health personnel, and psychological stress ^30^ . Infectious/transmissible diseases were mentioned in three studies ^25 , 26 , 38^ , and in a study conducted in Lebanon with 1,218 Syrian refugees, these diseases were prevalent (40.5%) ^38^ .

The vulnerability indicator “work” was found in three studies ^27 , 28 , 37^ . The unemployment rate among Syrian refugees was 84.2% in the study population ^28^ . The indicator “beliefs and values” concerning the disease was found in four studies ^23 , 28 , 29 , 35^ . Only 58% of Syrian women in Lebanon used some method of contraception; the birth rate is high, noting that women over 40 years old reported having between 8 and 12 children ^23^ .

The indicator “living conditions” was found in five studies ^17 , 20 , 22 , 32 , 56^ , disclosing insecurity, overcrowded housing, and absence of toilets in refuge camps ^17 , 22^ . More than half, 54.3%, reported the use of extreme mechanisms to face food insecurity (reduction of the portion; of the number of meals taken/day; reduced consumption by adults to allow feeding the children) ^20^ .

Regarding the programmatic dimension, the indicator “access to health services” was the most found in the studies (8) ^21^ , followed by the indicator “health policies” (7) ^22^ and the indicator “program actions” (6) ^21 , 29^ . Regarding access to health services, 60% of Syrian pregnant women in Turkey faced difficulties due to language and waiting time for care ^37^ . The lack of medical professionals and the cost ^21 , 23 , 24 , 38^ were reported as barriers to access for perinatal care ^22^ . In Greece, a study found that 50% of consultations in medical specialties were performed by national and international NGOs, unveiling a transfer of responsibility from the local government to these entities in providing access to health services. The main causes of referrals are obstetric/gynecological (35%) care, pediatric care (15%), and to obtain prescription drugs not available in PHC (16%) ^26^ .

Regarding the “Health Policies” indicator, the lack of a universal and free system stands out ^22 , 27 , 56^ . In a study with Syrian refugees in Jordan, 80% of respondents reported the need to pay some amount for health care in PHC ^22^ .

As for the indicator “actions of health programs”, included in the programmatic dimension, it is evident the difficulty of PHC teams in interacting with Syrian refugees ^34^ , especially in Lebanon. About 40% of respondents in Jordan reported discrimination by the healthcare team ^21^ . It should be considered that health professionals suffer the tension resulting from structural processes and fragile local governance, exposing them to the extremes of lack of human and material resources, and especially of knowledge about the refugee’s health issue ^56^ .

In the social dimension, the indicator “access to education” ^21 , 23 , 24 , 27 , 28 , 32 , 33 , 35 , 37 , 56^ was observed, and four of these studies ^21 , 24 , 28 , 37^ specifically approach with the difficulty of access to studies by the adult refugee population. In one of the studies, 42% had not completed any formal education ^35^ . A high rate (55%) of school dropouts by children was also found ^21^ . In Australia, a study reported that 25% of Syrian children were out of school even after three months in the country, and 67.9% had missed at least one school year before getting to the country ^33^ .

The indicator “access to information” was also observed ^21 , 23 , 24 , 35 , 37^ , identifying lack of information regarding the availability of family planning services in the Basic Healthcare Unit ( *Unidade Básica de Saúde,* UBS) ^21 , 23^ . One of the studies with Syrian refugees in Lebanon pointed out that 61% of the respondents were unaware of the availability of services ^21^ . On the other hand, international and local NGOs provide care for Syrian women through PHC, offering family planning free of charge (insertion of IUDs, contraceptive pills, and male condoms), as well as consultation with a midwife or obstetrician/gynecologist, and laboratory tests ^21^ .

The marker “migration” was found in three studies ^23 , 37 , 56^ . The pattern of migration flow affects the adherence to treatment of cardiovascular diseases in Syrian refugees arriving in Jordan, because during the trip they are unable to acquire the medicines ^29^ . In Jordan, a study indicated that 50% of the Syrians had concrete plans to leave the country, mainly towards Europe, due to the lack of access to fundamental rights, including education, health, work, and food. Part of one statement stands out: “ *a quick death at sea is better than dying a thousand deaths every day* ” ^56^ .

### II - Palestinian Refugees

Table 3 summarizes the 14 studies dealing with this refugee population ^39^ . Diabetes was the most noticeable disease ^40 , 42 , 43 , 45 , 48 , 50 , 52^ . In a study of 2,851 Palestinians aged up to 60 years and allocated in Jordan, the association between DM and SAH reaches 77% of the sample ^50^ , and obesity is one of the main additional associated risk factors. In another study, 59% of diabetic patients were obese (BMI > 30), 69% were women ^52^ . One of the surveys of Palestinian refugees in Jordan showed an association between DM and SAH in 81% of the sample, affecting all age groups: 21% were younger than 5 years old, 36% between five and 10 years old, and 43% were older than 10 years ^42^ . Hypertension also stands out among the main morbidities ^42 , 44 , 45 , 48 , 50 , 52^ , with increased prevalence from 2008 to 2012 among Palestinian refugees in Jordan (two million), ranging from 13.8% to 16.3% of the patients seen. Noteworthy are patients with uncontrolled hypertension, which, in the same period, ranged from 31.5% to 45.9% of patients ^44^ .

**Table 3:** Health vulnerabilities of Palestinian refugees.

Author/year	Type of study	Objective of the study	Population of the study	Vulnerabilities identified		

				Individual	Programmatic	Social
Al Baz, M. Law, M. R. Saadeh, R. ^39^ (2018)	Quantitative Cross-Sectional Study.	Study knowledge, attitudes and behaviors about antibiotic use.	250 Palestinians in Jordan. Mean: 32 years 205 women and 45 men over 18 years.	47% use of self-medication with antibiotics; Unemployment; Income below the national average.	Waiting hours in the health service.	-
Khader. A. et al. ^40^ (2014)	Quantitative Cohort.	Report the complications of diabetes in a cohort of refugees.	119 diabetic Palestinians in Jordan aged 0 to > 60 years.	Diabetes, obesity, sedentary lifestyle, cardiovascular disease. 57% had additional risk of obesity (BMI > 30).	Progressive loss in treatment follow-up (p < 0.001) Progressive reduction in proportion of patients attending clinic each year (p < 0.001)	-
Riccardo, Flavia; Khader, Ali; Sabatinelli, Guido ^41^ (2011)	Quantitative Cross-Sectional Study.	Analyze trends in infant mortality, among Palestinian refugees (1995-2005).	14,202 children in Jordan, Lebanon, Syria, Gaza Strip and West Bank.	Infant mortality rate higher than local population. Low Birth Weight/ Prematurity. Congenital malformation associated with early pregnancy and consanguineous marriage. Respiratory infections.	Poor access to health care: main condition for neonatal death. Fragmented care, highly privatized health system.	-
Khader. A. et al. ^42^ (2014)	Quantitative Cohort.	Determine the outcomes of diabetes mellitus (DM) treatment.	2,246 Palestinians in Jordan aged 0 to > 60 years with DM.	81%: Diabetes + SAH. 58%: DM controlled.	Difficulty of staff to maintain treatment follow-up.	
Al Kasseh, A. S. M. et al. ^43^ (2013)	Quantitative Retrospective case control.	Determine the risk factors for gestational diabetes in refugee women.	189 Palestinian Women in the Gaza Strip. 18 to 36 years/Median age 34.1 years.	Risk factors for Gestational Diabetes: history of spontaneous abortion (more than 1) (p < 0.0001); Weight before pregnancy (p < 0.001); Family history of DM (p < 0.001); History of stillbirth (p = 0.002); Weight in pregnancy (p < 0.001).	Weakness of preventive policies in the postpartum period.	Low educational level was a factor associated (p < 0.001) with Gestational Diabetes.
Saadeh. R. et al. ^44^ (2015)	Quantitative Unidentified Study Design.	Describe trends in use of antihypertensive drugs.	244,169 Palestinians in Jordan > 40 years.	Increase in prevalence of hypertension from 2008 to 2012.	Increase in program spending on antihypertensives drugs.	
Khader. A. et al. ^45^ (2014)	Quantitative Cohort.	Determine characteristics of refugees and the Hypertension program.	18,881 Palestinians in Jordan > 60 years old, with Hypertension	Hypertension, diabetes, obesity, congestive heart disease, sedentary lifestyle, acute myocardial infarction, stroke, and blindness.		
Abouteir. A. et al. ^46^ (2011)	Quantitative Prospective Case Control.	Investigate the relationship between diarrhea frequency and potential risk factors, including access to water.	3,338 Palestinians in the Gaza Strip. 266 patients with diarrhea, aged 0 to > 40 years.	Respiratory infection and diarrhea. Factors associated with diarrhea (p < 0.05): Socioeconomic status, poverty, unemployment, lack of access to public water, pets, poor sanitation.	Prescribed drug therapy does not follow WHO recommendations.	
Alameddine. M. et al. ^47^ (2019)	Qualitative Semi-structured Interview Thematic Analysis.	Understand the resilience of health systems.	61 Palestinians in Lebanon and Jordan; 37 in refugee camp/24 outside the camp;	PTSD, violence against women, depression.	Overloaded health care team, and high absenteeism rate. Stress in the work environment.	
Khader. A. et al. ^48^ (2012)	Quantitative Cohort.	Recording, monitoring and reporting chronic diseases.	4,130 Palestinians in Jordan with Hypertension from 0 to > 60 years.	Diabetes, cardiovascular disease, and hypertension.	Failure to measure blood pressure and blood glucose tests.	
Bastin. P. et al. ^49^ (2013)	Quantitative Cohort.	Identify mental diseases.	1,107 Palestinians, Iraqis and Lebanese - 18 to > 60 years.	28%: depression 15.6%: anxiety 11.5%: Psychoses.		Lack of access to education associated with mental health issues.
Khader. A. et al. ^50^ (2012)	Quantitative Cohort.	Assess the quality of services for refugees with diabetes.	2,851 Palestinians in Jordan with Diabetes from 0 to > 60 years.	Diabetes, blindness, stroke, cardiovascular disease, and amputations.	Failure to maintain treatment adherence after 12-month period. Failure of the health care team to follow protocols.	
Saleh. S. et al. ^51^ (2018)	Quantitative Randomized Controlled Clinical Trial.	Evaluate the effect of low-cost mobile health tools on accessibility to health services.	2,359 Palestinians in Lebanon 1,433 interventions 926 controls 40 to > 71 years old with DM and SAH.	Diabetes and hypertension.	The program showed weaknesses: maintaining annual eye checkup in patients with diabetes and hypertension, and in promoting smoking cessation. SMS did not generate clear intervention effect on the use of PHC services.	
Khader. A. et al. ^52^ (2013)	Quantitative Cohort.	Report the number and characteristics of new refugees with DM.	12. 550 Palestinians in Jordan with Diabetes from 0 to > 60 years.	Type I and II diabetes, hypertension, smoking, sedentary lifestyle, obesity. Complications of diabetes: acute myocardial infarction, end-stage renal disease.		

Regarding mental disorders, depression stands out (28%) ^47 , 49^ and among infectious diseases, diarrhea draws attention. One of the studies points out that 20.3% were infected with *Giardia duodenalis* , related to low water quality. The lack of access to free public drinking water was associated with infectious diarrhea, highlighting that 62.4% had to buy water ^46^ .

One of the studies, conducted in Jordan, Lebanon, Syria, Gaza, and the West Bank, identified the main causes of infant death in the first year of life: low weight and prematurity (30%), congenital malformation (22%), and respiratory tract infection (13.4%) ^41^ .

The indicator “work” was found in two studies ^39 , 46^ . The unemployment rate reaches 90% among Palestinian refugees in Jordan ^39^ . Even in the Palestinian territory itself, the majority of those who found employment had informal working relationships ^46^ .

The indicator “living conditions” was verified in two studies ^32 , 46^ . Palestinians live in the refugee camp for an average of 16.2 years, reaching up to 27.5 years, a much longer period than the Syrians. In this context, they live without public sanitation services, the houses/tents/containers are insufficient, and there is overcrowding ^32^ .

Regarding Programmatic Vulnerability, the indicator “actions of health programs” ^40 , 41 - 43 , 46 , 49 - 51^ was predominant. It is noteworthy the report of teams and patients that have mental and physical overload at work ^47^ . It is relevant to mention the loss of follow-up in the treatment of DM and SAH ^40 , 42 , 48 , 50^ . The increase in the migratory flow directly impacts the supply of services. In Jordan, of the patients who sought the PHC, 58% could not have their postprandial glycaemia measured ^50^ . Among patients with hypertension, 37% did not have their blood pressure recorded ^50^ , and 38% missed the scheduled return visit ^40^ .

The second most common indicator refers to “health policies” ^43 , 44 , 47^ mainly focused on drug intervention, with repercussions on spending on hypertension ^44^ . Only in 2011 did the UN introduce family health teams in Syria, Lebanon, and Jordan; previously, interventions were limited to medical centrality ^47^ .

Two studies addressed the Programmatic Vulnerability indicator “access to health services” ^39 , 41^ . In one, the majority (88%) of Palestinian refugees in Jordan reported waiting for long hours at the health facility, which resulted in doubling the risk of self-medication with antibiotics ^39^ . In the Gaza Strip and West Bank, poor access to PHC services was the main determinant of neonatal death ^41^ .

Regarding the elements of social vulnerability, the indicator most cited was “access to education” ^32 , 43 , 49^ . One of the studies points out that Palestinian refugees without any level of education in Lebanon accounted for 15.5% of the sample, almost double the local Lebanese population with the same level of education (8%) ^32^ . Low educational level among pregnant Palestinian refugees in the Gaza Strip was a factor associated with predisposition to gestational diabetes ^43^ . Studies confirm social exclusion in relation to participation in the economy with informal underemployment among this group of refugees. Legal restrictions in the host countries, such as Lebanon, corroborate the stigma about Palestinians.

### III - Iraqi Refugees

Table 4 summarizes the five studies about this refugee population ^53^ . The prevalence of infectious diseases, malnutrition, mental disorders, and diabetes stands out. In adults, the prevalence of DM was higher than in the local population, and almost twice as high in women ^54^ . Among children, we highlight vitamin D deficit; growth and nutritional problems; latent tuberculosis; neurological/metabolic and learning/behavioral disorders; delayed development; as well as exposure to trauma and separation from a family member ^33 , 53 , 54^ .

**Table 4:** Health vulnerabilities of Iraqi refugees.

Author/year	Type of study	Objective of the study	Population of the study	Vulnerabilities identified		

				Individual	Programmatic	Social
Worabo, H.J. et al. ^53^ (2016)	Qualitative In-depth focal group Phenomenological Analysis	Understand newly arrived refugees’ perceptions of the US healthcare system.	10 Iraqis (> 18 years old) in the US; 7 Men.	Infectious diseases, malnutrition, and mental health problems. Reluctance to adhere to latent TB treatment because they had no symptoms, and did not feel sick.	Language barriers caused abandonment of treatment. Lack of interpreter-translator. Team does not listen to complaints. Long wait for a consultation at the PHC.	They do not look for PHC because they cannot afford a translator, and due to the high cost of health services.
Goosen. S. et al. ^54^ (2014)	Descriptive Population Quantitative Study.	Map the prevalence and incidence of diabetes.	9,436 Iraqis in the Netherlands. 1,169 Syrians. Age Group: 20 to 79 years old.	Diabetes: twice as prevalent among refugees compared to the local population		
Van Loenen. T. et al. ^55^ (2018)	Qualitative Semi-structured Interview / Thematic Analysis.	Understand refugees’ health needs, barriers to access, and desires regarding PHC.	39 Syrians, 12 Iraqis in Greece, Slovenia, Croatia, Hungary, Netherlands, Italy, Austria.		Lack of mental health service provision in PHC. Lack of healthcare continuity. Difficulties in finding medical care at the busiest borders.	
Kvittingen. A. et al. ^56^ (2018)	Qualitative In-depth Interview.	Understand the experiences of Syrian and Iraqi refugees, and their migration aspirations.	62 refugees in Jordan: 32 Iraqis and 30 Syrians.	Lack of formal employment. None of the respondents were able to obtain legal residency.	Policies of revoking free access to public health and education, and limiting access to international humanitarian assistance.	50.0% of respondents had concrete plans to leave the country due to restrictions and lack of opportunities.
Doocy. S; Burnham G. ^57^ (2011)	Quantitative Unidentified Design.	Provide information on family economy and livelihoods of Iraqi refugees in urban area of Syria.	800 families of Iraqis in Syria.	Overcrowding of accommodations. Only 12% with stable work; heavy work; child labor; long work hours/week; underpayment or no payment for work done.	Unawareness about access to family planning services.	Poverty related to low educational status.

“Work” was another indicator checked ^53 , 56 , 57^ . In a study with Iraqi refugees in the United States, unemployment reached 90% ^53^ , with a predominance of informal work. The majority (61%) of Iraqi refugees in Syria hold casual jobs in the service sector (commerce), and only 12% have formal and stable jobs. Hard work is significant, with an average of 59 hours a week. There were also reports of child labor, underemployment, and payment for labor far below market rates, in addition to lack of payment after providing services ^56^ .

Regarding the “beliefs and values” indicator, the non-adherence to treatment for latent tuberculosis stands out. The lack of symptoms leads to the belief that treatment is unnecessary ^53^ .

Regarding the programmatic dimension of vulnerability, the indicator “actions of health programs” ^33 , 53 , 55^ stands out, mainly in the form of long waiting hours (in American health services), as well as lack of translator and empathy by the health team ^53^ . In one study, only 1.8% of the children had a complete health evaluation according to the protocol recommended for refugees ^33^ .

Regarding “access to healthcare services” ^33 , 55 , 57^ , difficulty to perform tuberculin skin test was observed among Iraqi refugees in the Australian PHC; and difficulty of access to health care in more crowded European borders ^55^ . Moreover, many patients did not use contraceptive methods, and 82% could not access them because they were unaware of the family planning services available ^57^ .

As for the “health policies” indicator, one study mentions that Jordan revoked the free access of Iraqi refugees to health care and fundamental rights, a step backwards in this free health care offer, motivated by the discourse of financial deficit ^56^ .

The most common indicator regarding the social dimension of vulnerability is “access to education” ^33 , 49 , 56 , 57^ . About 32% of Iraqi children in Australia missed three or more years of school in the pre-arrival period ^33^ . Children have difficulty enrolling in school because they lack an official residential address in Jordan ^56^ . Refugees with less education were more susceptible to mental illness ^49^ , poverty, and about twice as susceptible to a per capita income below $1/day ^57^ . The “migration” indicator brings up the sense of constant threat, for fear of expulsion by the government, experienced by Iraqi refugees in Jordan ^56^ .

## DISCUSSION

All dimensions of vulnerability were present in the studies of our sample. The bibliometric findings indicate that the predominance (77%) of quantitative studies may result in partial assessments of the life experiences of the populations studied. Studies conducted entirely in refugee camps and studies with significant samples in Brazil and Latin America were also scarce (8%), in addition to the scarcity of multicenter studies. There was no mention in the studies or comparative analysis regarding the difference in access to health care between refugees and asylum seekers. There was no study on comparative analysis of health vulnerabilities between Arab refugees and refugees of other nationalities. It is worth noting that “refugees” and “asylum seekers” are part of the same forced migration bloc; however, asylum seekers are only waiting for a bureaucratic step for their recognition as refugees in the host country.

Among Syrians, there is a lack of studies on the health needs of specific groups such as children, adolescents and women, and no studies on the health needs of female Palestinian refugee in PHC have been identified. Although Turkey is one of the main destination routes of Syrians, it was observed in our sample scarcity of studies (5%) related to the context of PHC in that host country.

Regarding the dimensions of vulnerability, the individual dimension reveals the presence of chronic and infectious diseases, besides the significant presence of mental disorders. These are corroborated by and attributed to traumatic experiences, even before forced migration: violence, abuse, and uncertainty about the future ^58^ . Despite the significance of mental disorders, this review and other studies point to a new challenge regarding the escalation of NCDs, with repercussions on the health system of the host countries in areas of conflict, especially Jordan, Lebanon, and Turkey, and increased spending on treatment, especially for DM and hypertension ^22 , 24 , 27 , 31 , 34 , 35 , 38 , 44 , 54 , 59^ . It is noteworthy that in the studies analyzed in this review, PHC played a central role in providing access to health systems in countries neighboring the conflict zones ^47 , 60^ .

Findings regarding women’s health are supported by a study that identified gestational diabetes, stillbirths, and children with low birth weight, in addition to inadequate prenatal care ^61^ . Congenital malformation was associated with early pregnancy and consanguineous marriage in Palestinian refugee women ^41^ . Refugee children suffer from conditions resulting from inadequate nutrition, malnutrition, micronutrient deficiencies, as well as oral health needs and infectious diseases, in addition to the consequences of exposure to war conflicts, violence, and xenophobia, and developmental delays and failures in schooling, among others ^62^ . Recent findings show evident inequality in vaccination coverage among refugee children when compared to the general population: they are three times less likely to be vaccinated against preventable infections, especially measles, tetanus, and meningitis C ^63^ . The difficulty in access to vaccines, and low vaccination coverage upon arrival in the host countries are evident in this study ^33^ .

Regarding the programmatic dimension, fragmentation of policies, programs, services, and health teams stands out. The PHC teams that work in the front line, in regions of large migration flows, have difficulties in dealing with refugees and work overload, besides absenteeism ^47^ . Another study supports and points out this phenomenon as a consequence of conflicts and war, which impact the entire local health system and that of neighboring countries, reducing the supply and quality of services, as well as promoting an exodus of health professionals. As a result, exclusionary policies that restrict and bureaucratize access to PHC have been adopted, such as more stringent document requirements for the acquisition of housing and food subsidies, end of gratuity, and beginning of charging for PHC services ^64^ . There is inequality in access, especially for refugees with lower educational level, who are more vulnerable among the vulnerable and depend on assistance from local governments ^17 , 24 , 27 , 32^ . A cross-sectional study of 400 Syrian refugees in Canada points out that refugees accessing public or local government-funded health care compared to refugees accessing private or privately funded health care report more unmet health needs and more complex medical conditions, and are almost three times more likely (OR = 2.84; 95%CI: 1.55–5.20) to not have their health needs met. Of those refugees, only 58% report having a family doctor of referral ^65^ . There are reports of a growing need for refugees in Lebanon and Jordan to pay for PHC services ^22 , 27 , 56^ . Although refugees disburse less than the local population, it is noteworthy that this situation is added with high unemployment, and low income (less than two dollars/day among refugees) ^22^ . The UN/UNHCR offers a program of income distribution and financial assistance for Syrian refugees in Jordan, for example, but only 23,000 families have access ^22^ to it. It is a very restricted number considering the more than 676,300 Syrian refugees under UN protection in that country ^4^ . In Jordan, a biometric personal identification card is required for access to health and nutritional support, but there are a number of requirements to obtain it ^17 , 22^ . As a result, many Syrians have been forced to further reduce their food intake, stop seeking health care, and take children out of school to offset costs or generate additional income through child labor ^17^ . The average number of meals for adult Syrian refugees in Lebanon was 1.8 meals/day, and among children, 2.3 meals/day ^17^ .

As for the main destination and host countries, Jordan, Lebanon and Syria, there is no explicit information on the websites of their respective Ministries of Health about public health policies for refugees. Lebanon is not a signatory to the 1951 Refugee Convention and, in this sense, there is no domestic law addressing refugee needs in the country ^68^ . There are records that Palestinians and Syrians suffer from marginalization and discrimination as a result of policies that deny access to basic rights such as housing, work, education and health care ^69^ . The United Nations Relief and Works Agency (UNRWA) has suffered major budget cuts, and its largest donor, the United States, has cut funding ^69^ . However, there is controversy about governmental actions regarding refugees in this country, pointing out the existence of primary health care provision, which includes consultations, lab and diagnostic testing for groups previously defined as vulnerable, offered at a reduced cost to residents ^69^ . Vaccination, two ultrasound examinations for pregnant women, and medication for acute and chronic conditions are free of charge ^69^ .

In Jordan, it is noted that rental housing is affordable, and housing in settlements is offered in exchange for work on local farms ^69^ . The reduced offer of labor determines the need to work or get married, which leads to dropping out school ^69^ . To mitigate such situations, the government has implemented a program: “Cash+”, which includes social protection interventions for families in vulnerable situations, who get unconditional monthly cash transfer per child. Mental health programs are also offered in integration with primary health care services ^69^ . Refugees in camps have free access to health care, subsidized by the government and international agencies. Refugees registered with the Ministry of Interior in Jordan have access to healthcare and government benefits, in the same way as uninsured Jordanians. However, it is noteworthy that refugees have financial burden to afford with consultations and medication in private health services ^70^ .

As for the social dimension of vulnerabilities, inequality was evidenced in the access to education, information, decent work, besides prejudice/stigma, and difficulties of participation in collective actions and consequent difficulty of integration in the host countries ^17 , 21 - 24 , 27 , 32 , 33 , 35 , 37 , 43 , 49^ . Indeed, social disconnection and comorbidities are prevalent, and lack of engagement in the community was associated with unfavorable health outcomes, especially in relation to mental health. Difficult social integration persisted for three or more decades after arrival in the US, constituting a health risk factor ^66^ . Other findings point to the social exclusion of refugees as a consequence of structural inequalities, including marginalizing policies, and lack of social security. The lack of basic services in the host countries leads to disputes between local population and refugees, besides the progressive degradation of living conditions ^67^ .

Regarding the recommendations for policies and practices in PHC, there is a need for screening cases and strengthening programs and policies, especially in the field of mental health, with Syrian and Palestinian refugees ^36^ . Evidence suggests the indication of diabetes screening for newly arrived refugees older than 35 years ^54^ . Healthcare providers should be aware of the high risk for diabetes among Syrian, Palestinian, and Iraqi refugees ^54^ . Moreover, there is a need to strengthen women’s health programs, aiming at early identification of gestational diabetes, including in the postpartum period, especially among Palestinian refugees ^43^ . In order to improve adherence and success in the treatment of tuberculosis among refugees, there is a need for a specific program for the group mainly addressing beliefs about the disease and the treatment of latent tuberculosis ^30^ .

One of the limitations of this research is the restriction to studies with subjects who forcibly migrated; therefore, the vulnerabilities mapped are not generalizable to the bloc of non-forced migrations. The vulnerability elements found do not encompass the context of the 22 Arab countries in the world, being restricted to Syrians, Palestinians, and Iraqis. It is also important to point out the heterogeneity of the methods used, which made the process of data extraction difficult, even when the study dealt with refugees from more than one country of Arab origin, because some did not separate outcomes by nationality, which made the extraction and synthesis of this review difficult.

## CONCLUSION

The Asian continent, the Middle East, followed by North America and the European Union produced the most studies with Arab refugees in the context of PHC. There is an evident gap in the production of knowledge about this subject in the Latin American continent, and none of the studies used the concept of vulnerability, as adopted in this scoping review.

Arab refugees experience contexts of high vulnerability, placing them in profound inequality and disadvantage before the health programs, services and system of the host country. Therefore, there is a need for programs and policies that consider the elements of vulnerability and promote actions in PHC in order to respond to the health needs of refugees. It should be considered that the clash of forces between countries, mainly due to economic interests, causes destructive repercussions to populations, imposing its urgent overcoming and intransigence, besides the repudiation of actions that show social injustice. We defend the need for policies of inclusion, social justice and dignified living conditions for all people in situations of refuge, rejecting all kinds of stigmatizing attitudes and practices that show dehumanization.

## References

[1] International Organization for Migration . World Migration Report 2020 . Geneva (CH) : IOM ; 2019 [ cited 2020 Oct 21 ]. Part I, Data and information on migration and migrants; Chapter 2, Migration and migrants: a global overview ; p. 19 - 52 . Available from: https://publications.iom.int/system/files/pdf/wmr_2020.pdf

[2] United Nations High Commissioner for Refugees . Global trends: forced displacement in 2019 . Geneva (CH) : UNHCR ; 2020 [ cited 2020 Oct 21 ]. Available from: https://www.unhcr.org/statistics/unhcrstats/5ee200e37/unhcr-global-trends-2019.html

[3] Alto Comissariado das Nações Unidas para Refugiados . Protegendo refugiados no Brasil e no mundo . São Paulo, SP : ACNUR ; 2016 [ cited 2020 Oct 3 ]. Available from: https://www.acnur.org/portugues/

[4] United Nations High Commissioner for Refugees . Global trends: forced displacement in 2018 . Geneva : UNHCR ; 2019 [ cited 2020 Oct 21 ]. Available from: https://www.unhcr.org/5d08d7ee7.pdf

[5] United Nations High Commissioner for Refugees . Global trends: forced displacement in 2016 . Geneva (CH) : UNHCR ; 2017 [ cited 2020 Oct 12 ]. Available from: https://www.unhcr.org/statistics/unhcrstats/5943e8a34/global-trends-forced-displacement-2016.html

[6] Calegari M . Condições de vida dos refugiados sírios em São Paulo . In: Baeninger R , Bógus LM , Moreira JB , Vedovato LR , Fernandes DM , Souza MR , et al , organizadores . 2 . ed. Campinas, SP : Nepo/Unicamp ; 2018 . p. 325 - 38 .

[7] Coutts A , Mckee M , Stuckler D . The emerging Syrian health crisis . Lancet . 2013 ; 381 ( 9865 ): e6 - 7 . 10.1016/S0140-6736(13)60053-7 23375240

[8] Ozaras R , Balkan II , Yemisens M . Prejudice and reality about infection risk among Syrian refugees . Lancet . 2016 ; 16 ( 11 ): 1222 - 3 . 10.1016/S1473-3099(16)30400-5 27788978

[9] Ministério da Justiça (BR) , Comitê Nacional para Refugiados . Sistema de Refúgio Brasileiro: desafios e perspectivas. Refúgio em números . Brasília, DF : CONARE ; 2017 [ cited 2020 Oct 20 ]. Available from: https://www.acnur.org/fileadmin/Documentos/portugues/Estatisticas/Sistema_de_Refugio_brasileiro_-_Refugio_em_numeros_-_05_05_2016.pdf

[10] Ministério da Justiça (BR) , Comitê Nacional para Refugiados . Refúgio em números . Brasília, DF : CONARE ; 2018 [ cited 2020 Oct 15 ]. Available from: https://www.acnur.org/portugues/wp-content/uploads/2018/04/refugio-em-numeros_1104.pdf

[11] World Health Organization . 2017. Vulnerability and resilience; Sri Lanka . Geneva (CH) : WHO ; 2017 [ cited 2020 Oct 1 ]. Available from: https://www.iom.int/sites/g/files/tmzbdl486/files/our_work/DMM/Migration-Health/GC2_TDP_Vulnerability-and-Resilience_FINAL_13.02.2017.pdf

[12] Arksey H , O’Malley L . Scoping studies: towards a methodological framework . Int J Soc Res Methodol . 2005 ; 8 ( 1 ): 19 - 32 . 10.1080/1364557032000119616

[13] Ayres JRCM , Paiva V , França Jr I . Conceitos e práticas de prevenção: da história natural da doença ao quadro da vulnerabilidade e direitos humanos . In: Paiva V , Ayres JRCM , Buchalla CM , organizadores . Vulnerabilidade e direitos humanos: prevenção e promoção da saúde: Livro 1, Doença e cidadania . Curitiba, PR : Juruá ; 2012 . p. 71 - 94 .

[14] Ayres JRCM , Calazans GJ , Saletti Filho HC , França Júnior l . Risco, vulnerabilidade e práticas de prevenção e promoção da saúde . In: Campos GWS , organizador . Tratado de Saúde Coletiva . São Paulo : Hucitec; Fiocruz ; 2006 . p. 375 - 417 .

[15] Moher D , Liberati A , Tetzlaff J , Altman DG ; The PRISMA Group . Preferred reporting items for systematic reviews and meta-analyses: the PRISMA Statement . PLoS Med . 2009 ; 6 ( 7 ): e1000097 . 10.1371/journal.pmed.1000097 PMC2707599 19621072

[16] Nichiata LYI , Takahashi RF , Bertolozzi MR . Perspectivas avaliativas das vulnerabilidades em saúde . In: Egry EY , organizadora . Necessidades em saúde na perspectiva da Atenção Básica: guia para pesquisadores . São Paulo : Dedone , 2008 . p. 41 - 47

[17] United Nations High Commissioner for Refugees ; UNICEF ; WFP . Vulnerability assessment of Syrian refugees in Lebanon 2016 . Geneva (CH) : UNHCR ; 2016 .

[18] Hosten E , Mehta M , Andre E , Abu Rumman KA , Van der Linden D . Tuberculosis contact-tracing among Syrian refugee populations: lessons from Jordan . Confl Health . 2018 ; 12 : 25 . 10.1186/s13031-018-0164-y PMC6047119 30026793

[19] Truppa C , Leresche E , Fuller AF , Marnicio AS , Abisaab J , El Hayek N , et al . Utilization of primary health care services among Syrian refugee and Lebanese women targeted by the ICRC program in Lebanon: a cross-sectional study . Confl Health . 2019 ; 13 : 7 . 10.1186/s13031-019-0190-4 PMC6420751 30923560

[20] Doocy S , Lyles E . Humanitarian needs among displaced and female-headed households in government-controlled areas of Syria . Am J Public Health . 2017 ; 107 ( 6 ): 950 - 9 . 10.2105/AJPH.2017.303710 PMC5425854 28426308

[21] Ay M , Arcos González P , Castro Delgado R . The perceived barriers of access to health care among a group of non-camp Syrian refugees in Jordan . Int J Health Serv . 2016 ; 46 ( 3 ): 566 - 89 . 10.1177/0020731416636831 26962004

[22] Al-Rousan T , Schwabkey Z , Jirmanus L , Nelson BD . Health needs and priorities of Syrian refugees in camps and urban settings in Jordan: perspectives of refugees and health care providers . East Mediterr Health J . 2018 ; 24 ( 3 ): 243 - 53 . 10.26719/2018.24.3.243 29908019

[23] Cherri Z , Cuesta JG , Rodriguez-Llanes JM , Guha-Sapir D . Early marriage and barriers to contraception among Syrian refugee women in Lebanon: a qualitative study . Int J Environ Res Public Health . 2017 ; 14 ( 8 ): 836 . 10.3390/ijerph14080836 PMC5580540 28757595

[24] Doocy S , Lyles E , Roberton T , Akhu-Zaheya L , Oweis A , Burnham G . Prevalence and care-seeking for chronic diseases among Syrian refugees in Jordan . BMC Public Health . 2015 ; 15 : 1097 . 10.1186/s12889-015-2429-3 PMC4628338 26521231

[25] Gammoh OS . A preliminary description of medical complaints and medication consumption among 375 Syrian refugees residing in North Jordan . Jordan J Pharm Sci . 2016 ; 9 ( 1 ): 13 - 21 .

[26] Kakalou E , Riza E , Chalikias M , Voudouri N , Vetsika A , Tsiamis C , et al . Demographic and clinical characteristics of refugees seeking primary healthcare services in Greece in the period 2015-2016: a descriptive study . Int Health . 2018 ; 10 ( 6 ): 421 - 9 . 10.1093/inthealth/ihy042 29992276

[27] Sethi S , Jonsson R , Skaff R , Tyler F . Community-based noncommunicable disease care for Syrian refugees in Lebanon . Glob Health Sci Pract . 2017 ; 5 ( 3 ): 495 - 506 . 10.9745/GHSP-D-17-00043 PMC5620345 28928227

[28] Aoun A , Joundi J , El Gerges N . Prevalence and correlates of a positive screen for eating disorders among Syrian refugees . Eur Eat Disord Rev . 2019 ; 27 ( 3 ): 263 - 73 . 10.1002/erv.2660 30549173

[29] Collins DRJ , Jobanputra K , Frost T , Muhammed S , Ward A , Shafei AA , et al . Cardiovascular disease risk and prevention amongst Syrian refugees: mixed methods study of Medecins Sans Frontieres programme in Jordan . Confl Health . 2017 ; 11 : 14 . 10.1186/s13031-017-0115-z PMC5512828 28725259

[30] Dogru S , Doner P . Frequency and outcomes of new patients with pulmonary tuberculosis in Hatay province after Syrian civil war . Indian J Tuberc . 2017 ; 64 ( 2 ): 83 - 8 . 10.1016/j.ijtb.2016.11.034 28410703

[31] Doocy S , Lyles E , Hanquart B ; LHAS Study Team , Woodman M . Prevalence, care-seeking, and health service utilization for non-communicable diseases among Syrian refugees and host communities in Lebanon . Confl Health . 2016 ; 10 : 21 . 10.1186/s13031-016-0088-3 PMC5070168 27777613

[32] Segal SP , Khoury VC , Salah R , Ghannam J . Contributors to screening positive for mental illness in Lebano´s Shatila Palestinian Refugee Camp . J Nerv Ment Dis . 2018 ; 206 ( 1 ): 46 - 51 . 10.1097/NMD.0000000000000751 28976407

[33] Heenan RC , Volkman T , Stokes S , Tosif S , Graham H , Smith A , et al . ‘I think we’ve had a health screen’: new offshore screening, new refugee health guidelines, new Syrian and Iraqi cohorts: recommendations, reality, results and review . J Paediatr Child Health . 2019 ; 55 ( 1 ): 95 - 103 . 10.1111/jpc.14142 30094942

[34] Doocy S , Paik KE , Lyles E , Hei Tam H , Fahed Z , Winkler E , et al . Guidelines and mHealth to improve quality of hypertension and type 2 diabetes care for vulnerable populations in Lebanon: longitudinal cohort study . JMIR Mhealth Uhealth . 2017 ; 5 ( 10 ): e158 . 10.2196/mhealth.7745 PMC5695979 29046266

[35] Elliott JA , Das D , Cavailler P , Schneider F , Shah M , Ravaud A , et al . A cross-sectional assessment of diabetes self-management, education and support needs of Syrian refugee patients living with diabetes in Bekaa Valley Lebanon . Confl Health . 2018 ; 12 : 40 . 10.1186/s13031-018-0174-9 PMC6134700 30214472

[36] Javanbakht A , Amirsadri A , Suhaiban HA , Alsaud MI , Alobaidi Z , Rawi Z , et al . Prevalence of possible mental disorders in Syrian refugees resettling in the United States screened at primary care . J Immigr Minor Health . 2019 ; 21 ( 3 ): 664 - 7 . 10.1007/s10903-018-0797-3 30066059

[37] Torun P , Karaaslan MM , Sandikli B , Acar C , Shurtleff E , Dhrolia S , et al . Health and health care access for Syrian refugees living in Istanbul . Int J Public Health . 2018 ; 63 ( 5 ): 601 - 8 . 10.1007/s00038-018-1096-4 29629476

[38] Lyles E , Hanquart B , Chlela L , Woodman M ; LHAS Study Team , Fouad FM , Sibai A , et al . Health service access and utilization among Syrian refugees and affected host communities in Lebanon . J Refugee Stud . 2018 ; 31 ( 1 ): 104 - 30 . 10.1093/jrs/fex014

[39] Al Baz M , Law MR , Saadeh R . Antibiotics use among Palestine refugees attending UNRWA primary health care centers in Jordan: a cross-sectional study . Travel Med Infect Dis . 2018 ; 22 : 25 - 9 . 10.1016/j.tmaid.2018.02.004 29458088

[40] Khader A , Ballout G , Shahin Y , Hababeh M , Farajallah L , Zeidan W , et al . Treatment outcomes in a cohort of Palestine refugees with diabetes mellitus followed through use of E-Health over 3 years in Jordan . Trop Med Int Health . 2014 ; 19 ( 2 ): 219 - 23 . 10.1111/tmi.12241 24341942

[41] Riccardo F , Khader A , Sabatinelli G . Low infant mortality among Palestine refugees despite the odds . Bull World Health Organ . 2011 ; 89 ( 4 ): 304 - 11 . 10.2471/BLT.10.082743 PMC3066522 21479095

[42] Khader A , Ballout G , Shahin Y , Hababeh M , Farajallah L , Zeidan W , et al . What happens to Palestine refugees with diabetes mellitus in a primary healthcare centre in Jordan who fail to attend a quarterly clinic appointment? Trop Med Int Health . 2014 ; 19 ( 3 ): 308 - 12 . 10.1111/tmi.12256 24387037

[43] Alkasseh ASM , Zaki NM , Aljeesh YI , Soon LK . Risk factors of gestational diabetes mellitus in the refugee population in Gaza Strip: a case-control study . East Mediterr Health J . 2014 ; 19 Suppl 3 : S12 - 8 . 24995734

[44] Saadeh R , Qato D , Khader A , Shahin Y , Seita A . Trends in the utilization of antihypertensive medications among Palestine refugees in Jordan, 2008-2012 . J Pharm Policy Pract . 2015 ; 8 : 7 . 10.1186/s40545-015-0036-4 PMC4436138 25992295

[45] Khader A , Farajallah L , Shahin Y , Hababeh M , Abu-Zayed I , Zachariah R , et al . Hypertension and treatment outcomes in Palestine refugees in United Nations Relief and Works Agency primary health care clinics in Jordan . Trop Med Int Health . 2014 ; 19 ( 10 ): 1276 - 83 . 10.1111/tmi.12356 25039838

[46] Abouteir A , El Yaagoubi F , Bioh-Johnson I , Kamel A , Godard N , Cormerais L , et al . Water access and attendance for diarrhea in primary health care centers, Gaza strip . Trans R Soc Trop Med Hyg . 2011 ; 105 ( 10 ): 555 - 60 . 10.1016/j.trstmh.2011.07.002 21803391

[47] Alameddine M , Fouad FM , Diaconu K , Jamal Z , Lough G , Witter S , et al . Resilience capacities of health systems: accommodating the needs of Palestinian refugees from Syria . Soc Sci Med . 2019 ; 220 : 22 - 30 . 10.1016/j.socscimed.2018.10.018 30390471

[48] Khader A , Farajallah L , Shahin Y , Hababeh M , Abu-Zayed I , Kochi A , et al . Cohort monitoring of persons with hypertension: an illustrated example from a primary healthcare clinic for Palestine refugees in Jordan . Trop Med Int Health . 2012 ; 17 ( 9 ): 1163 - 70 . 10.1111/j.1365-3156.2012.03048.x 22845700

[49] Bastin P , Bastard M , Rossel L , Melgar P , Jones A , Antierens A . Description and predictive factors of individual outcomes in a refugee camp based mental health intervention (Beirut, Lebanon) . PLoS One . 2013 ; 8 ( 1 ): e54107 . 10.1371/journal.pone.0054107 PMC3547969 23349795

[50] Khader A , Farajallah L , Shahin Y , Hababeh M , Abu-Zayed I , Kochi A , et al . Cohort monitoring of persons with diabetes mellitus in a primary healthcare clinic for Palestine refugees in Jordan . Trop Med Int Health . 2012 ; 17 ( 12 ): 1569 - 76 . 10.1111/j.1365-3156.2012.03097.x 23051859

[51] Saleh S , Farah A , Dimassi H , El Arnaout N , Constantin J , Osman M , et al . Using mobile health to enhance outcomes of noncommunicable diseases care in rural settings and refugee camps: randomized controlled trial . JMIR Mhealth Uhealth . 2018 ; 6 ( 7 ): e137 . 10.2196/mhealth.8146 PMC6064041 30006326

[52] Khader A , Ballout G , Shahin Y , Hababeh M , Farajallah L , Zeidan W , et al . Diabetes mellitus and treatment outcomes in Palestine refugees in UNRWA primary health care clinics in Jordan . Public Health Action . 2013 ; 3 ( 4 ): 259 - 64 . 10.5588/pha.13.0083 PMC4463155 26393043

[53] Worabo HJ , Kuei-Hsiang H , Yakimo R , Worabo E , Burgess PA , Farberman SM . Understanding refugees’ perceptions of health care in the United States . J Nurse Pract . 2016 ; 12 ( 7 ): 487 - 94 . 10.1016/j.nurpra.2016.04.014

[54] Goosen S , Middelkoop B , Stronks K , Agyemang C , Kunst AE . High diabetes risk among asylum seekers in The Netherlands . Diabet Med . 2014 ; 31 ( 12 ): 1532 - 41 . 10.1111/dme.12510 24860962

[55] Loenen T , Muijsenbergh M , Hofmeester M , Dowrick C , Ginneken N , Mechili EA , et al . Primary care for refugees and newly arrived migrants in Europe: a qualitative study on health needs, barriers and wishes . Eur J Public Health . 2018 ; 28 ( 1 ): 82 - 7 . 10.1093/eurpub/ckx210 29240907

[56] Kvittingen A , Valenta M , Tabbara H , Baslan D , Berg B . The conditions and migratory aspirations of Syrian and Iraqi refugees in Jordan . 2019 ; 32 ( 1 ): 106 - 24 . 10.1093/jrs/fey015

[57] Doocy S , Burnham G , Biermann E , Tileva M . Household economy and livelihoods among Iraqi refugees in Syria . J Refugee Stud . 2012 ; 25 ( 2 ): 282 - 300 . 10.1093/jrs/fer049

[58] Patel K , Kouvonen A , Close C , Väänänen A , O Reilly D , Donnelly M . What do register-based studies tell us about migrant mental health? A scoping review . Syst Rev . 2017 ; 6 ( 1 ): 78 . 10.1186/s13643-017-0463-1 PMC5387245 28399907

[59] Naja F , Shatila H , El Koussa M , Meho L , Ghandour L , Saleh S . Burden of non-communicable diseases among Syrian refugees: a scoping review . BMC Public Health . 2019 ; 19 : 637 . 10.1186/s12889-019-6977-9 PMC6534897 31126261

[60] Kruk ME , Ling EJ , Bitton A , Cammett M , Cavanaugh K , Chopra M , et al . Building resilient health systems: a proposal for a resilience index . BMJ . 2017 ; 357 : j2323 . 10.1136/bmj.j2323 28536191

[61] Liu C , Ahlberg M , Hjern A , Stephansson O . Perinatal health of refugee and asylum-seeking women in Sweden 2014-17: a register-based cohort study . Eur J Public Health . 2019 ; 29 ( 6 ): 1048 - 55 . 10.1093/eurpub/ckz120 PMC6896976 31274154

[62] Kroening ALH , Dawson-Hahn E . Health considerations for immigrant and refugee children . Adv Pediatr . 2019 ; 66 : 87 - 110 . 10.1016/j.yapd.2019.04.003 31230701

[63] Perry M , Townson M , Cottrell S , Fagan L , Edwards J , Saunders J , et al . Inequalities in vaccination coverage and differences in follow-up procedures for asylum-seeking children arriving in Wales, UK . Eur J Pediatr . 2020 ; 179 : 171 - 5 . 10.1007/s00431-019-03485-7 31701239

[64] Lafta RK , Al-Nuaimi MA . War or health: a four-decade armed conflict in Iraq . Med Confl Surviv . 2019 ; 35 ( 3 ): 209 - 26 . 10.1080/13623699.2019.1670431 31597450

[65] Oda A , Hynie M , Tuck A , Agic B , Roche B , McKenzie K . Differences in self-reported health and unmet health needs between government assisted and privately sponsored Syrian refugees: a cross-sectional survey . J Immigr Minor Health . 2019 ; 21 ( 3 ): 439 - 42 . 10.1007/s10903-018-0780-z 29959652

[66] Berthold SM , Loomis AM , Kuoch T , Scully M , Hin-McCormick MM , Casavant B , et al . Social disconnection as a risk factor for health among Cambodian refugees and their offspring in the United States . J Immigr Minor Health . 2019 ; 21 ( 2 ): 290 - 8 . 10.1007/s10903-018-0760-3 29796964

[67] Jirmanus LZ , Ziadee M , Usta J . Confronting structural inequities: the limits of participation when developing a community health intervention with Syrian refugees and host communities in Lebanon . Soc Sci Med . 2021 ; 272 : 113699 . 10.1016/j.socscimed.2021.113699 33556814

[68] United Nations High Commissioner for Refugees . UNHCR Global Appeal 2014-2015 . Geneva (CH) : UNHCR ; 2016 [ cited 2021 May 13 ]. www.unhcr.org./528a0a2da.pdf

[69] Majzoub A . Lebanon´s refugee restrictions could harm everyone´s health . New York : Human Rights Watch ; 2020 [ cited 2021 May 28 ]. Available from: https://www.hrw.org/news/2020/04/22/lebanons-refugee-restrictions-could-harm-everyones-health

[70] Dator W , Abunab H , Dao-Ayen N . Health challenges and access to health care among Syrian refugees in Jordan: a review . East Mediterr Health J . 2018 ; 24 ( 7 ): 680 - 6 . 10.26719/2018.24.7.680 30215478

