# Supplementary material for: Vulnerabilities of Arab refugees in primary health care: a scoping review
Source: Rev Saude Publica. 2022 Mar 23;56:15. doi: 10.11606/s1518-8787.2022056003691 (PMC8963749; doi:10.11606/s1518-8787.2022056003691)
Supplement: Translation

# Translation: Vulnerabilidades de refugiados árabes na atenção primária à saúde: uma revisão de escopo

**Authors:** Luiz Paulo de Lima, Kayte Chaves Oliveira de Lima, Maria Rita Bertolozzi, Francisco Oscar de Siqueira França

**Affiliations:** I Brasil Universidade de São Paulo. Faculdade de Saúde Pública. Programa de Pós-Graduação em Saúde Global e Sustentabilidade. São Paulo, SP, Brasil; II Brasil Universidade de São Paulo. Escola de Enfermagem. Programa de Pós-Graduação em Enfermagem. São Paulo, SP, Brasil; III Brasil Universidade de São Paulo. Escola de Enfermagem. Departamento de Enfermagem em Saúde Coletiva. São Paulo, SP, Brasil; IV Brasil Universidade de São Paulo. Faculdade de Medicina. Departamento de Moléstias Infecciosas e Parasitárias. Núcleo de Medicina Tropical. São Paulo, SP, Brasil

**Keywords:** Refugiados, Árabes, Necessidades e Demandas de Serviços de Saúde, Atenção Primária à Saúde, Vulnerabilidade em Saúde, Revisão

## Abstract

**OBJETIVO:**

Mapear e analisar as vulnerabilidades de refugiados árabes no contexto da atenção primária à saúde.

**MÉTODO:**

Revisão de escopo em que foram analisados estudos publicados nos idiomas inglês, espanhol e português, a partir de 2011. As bases foram Cochrane, Scopus, *Health System Evidence* , MedLine-PubMed, CINAHL, Embase, Lilacs, *Web of Science* , SciELO, NYAM *Grey Literature* , BVS, Banco de teses e dissertações Capes, *Refworld e Journal of Refugee Studies.* A análise dos dados foi realizada à luz do conceito de vulnerabilidade.

**RESULTADOS:**

Dos 854 estudos identificados, restaram 40 artigos para análise e extração dos indicadores de vulnerabilidade nas dimensões individual, social e programática. Em relação à dimensão individual, os principais indicadores identificados foram desemprego, moradias instáveis e superlotadas, falta de saneamento e de acesso à água, agravos mentais, doenças transmissíveis e crônicas não transmissíveis etc. Na dimensão programática foram identificadas, principalmente, equipes de saúde com sobrecarga de trabalho, falta de preparo para lidar com as barreiras culturais e linguísticas, demora para o atendimento. Em relação à dimensão social, constatou-se falta de acesso às escolas, à informação sobre os programas de saúde dos países de acolhida, aos direitos, entre outros.

**CONCLUSÃO:**

As vulnerabilidades constatadas evidenciam desvantagem dos refugiados perante os programas, serviços e sistema de saúde nos países de acolhida, além de colocar em evidência as profundas desigualdades que incidem nesse grupo. Aponta-se a necessidade de programas e políticas que promovam ações, no âmbito da atenção primária à saúde, que reconheçam e respondam às necessidades de saúde de refugiados.

## INTRODUÇÃO

Em 2019 estimava-se que 275 milhões de pessoas migraram para fora das fronteiras de seus próprios países, o que significa que 3,5% da população mundial eram migrantes internacionais. Esse número triplicou nos últimos 45 anos ^1^ . Atualmente, há cerca de 79,5 milhões de pessoas em deslocamento forçado e, desses, 26 milhões são refugiados ^2^ , pessoas que “estão fora de seu país natal devido a fundados temores de perseguição relacionados a conflitos armados, questões de raça, religião, nacionalidade, pertencimento a grupo social ou opinião política, como também devido à violação grave e generalizada de direitos humanos” ^3^ .

Em 2011, devido ao que se denominou primavera árabe e ao conflito armado na Síria, o mundo árabe passou a ser o epicentro na temática do refúgio. A Síria tem, hoje, a maior população em deslocamento forçado no mundo, com 13,5 milhões em 2019, 6,7 milhões deles são refugiados. O Iraque tem cerca de 3,3 milhões de pessoas nessa situação e a Palestina, 5,5 milhões ^2^ . O Líbano possui a maior concentração de refugiados no mundo em relação à população geral (156/1.000) ^4^ , o que agrava as condições de saúde no país, destacando-se que a maioria dos refugiados procedem da Síria ^5^ .

As tendências globais do refúgio repercutem também no Brasil. Desde o início do conflito na Síria, 3.772 pessoas solicitaram refúgio no país ^9^ e, entre os árabes, a Síria, Palestina, Líbano e Iraque são os países de origem que mais tiveram sua condição de refugiados reconhecida no Brasil ^10^ .

A condição de refúgio já é um elemento de vulnerabilidade, dada a migração forçada e por representar grave e generalizada violação de direitos humanos ^11^ . O presente artigo tem como objetivo mapear e analisar os elementos de vulnerabilidade em saúde de refugiados árabes no âmbito da atenção primária em saúde (APS).

## MÉTODO

Trata-se de uma revisão de escopo, conduzida segundo Arksey e O`Malley ^12^ em 5 estágios:

### Estágio I: Identificação da pergunta de pesquisa.

Quais são os elementos de vulnerabilidade, nas dimensões individual, programática e social, que incidem sobre os refugiados árabes no contexto da atenção primária à saúde?

### Estágio II: Identificação de estudos relevantes.

Foram utilizadas duas estratégias ( Tabela 1 ), adaptadas conforme a especificidade de cada base. As bases de dados foram: Lilacs, SciELO, NYAM *Grey Literature* , Cochrane, *Health System Evidence* , MedLine/PubMed, Embase, *Web of Science* , CINAHL, BVS, Banco de Teses e Dissertações Capes, Refworld e *Journal of Refugee Studies* . Foram incluídos estudos publicados entre 1 de janeiro de 2011 até 31 de dezembro de 2019, considerando o contexto histórico da primavera árabe.

**Tabela 1:** Estratégias de busca com descritores (MeSH) e palavras-chave.

**Descritores e palavras-chave para estratégia de busca 1** “Primary Health Care” [Keyword, MeSH]; OR “Primary Care” [Keyword]; OR “General Practice” [Keyword, MeSH]; OR “FamilyPractice” [Keyword, MeSH]; AND Refugee* [Keyword, MeSH]; OR “Asylum Seeker” [Keyword]; AND Arab [Keyword]; OR Syria [MeSH, Keyword]; OR Syrian [Keyword]; OR Palestinian [Keyword]; OR Palestine [Keyword]; OR Iraqi [Keyword]; OR Iraq [Keyword, MeSH]; OR Lebanese [Keyword]; OR Lebanon [Keyword, MeSH].
**Descritores e palavras-chave para estratégia de busca 2** Vulnerability [Keyword]; AND Arab [Keyword]; OR Syria [MeSH, Keyword]; OR Syrian [Keyword]; OR Palestinian [Keyword]; OR Palestine [Keyword]; OR Iraqi [Keyword]; OR Iraq [MeSH, Keyword]; OR Lebanese [Keyword]; ORLebanon [Keyword, MeSH].

### Estágio III: Seleção dos estudos.

Foram critérios de inclusão: sujeitos dos estudos eram refugiados árabes, sírios, palestinos, iraquianos e libaneses na condição decorrente de migração forçada, pois são as nacionalidades que mais tiveram o *status* de refugiado reconhecido no Brasil ^10^ ; estudos que possibilitavam identificar elementos constitutivos do conceito de vulnerabilidade; estudos que apresentavam elementos de vulnerabilidade em qualquer das quatro fases do refúgio (origem, trânsito, destino e retorno); estudos que tiveram como cenário a APS.

Foram excluídos os estudos: sobre migrações voluntárias (deslocamento não forçado e migração econômica); não traziam dados originais/primários (artigo de opinião, relatos de experiência, revisões de literatura). Não delimitamos o tempo de migração dos sujeitos dos estudos.

Nós consideramos vulnerabilidade “um conjunto de aspectos individuais e coletivos relacionados à maior suscetibilidade de indivíduos e comunidades a um adoecimento ou agravo e, de modo inseparável, a menor disponibilidade de recursos para sua proteção” ^13^ . Também consideramos as três dimensões de análise interdependentes, inextricáveis e sinérgicas do conceito: individual, programática e social ^13^ . A dimensão individual toma como ponto inicial aspectos próprios ao modo de vida, que podem contribuir para a exposição a um determinado agravo à saúde, ou aspectos que podem proteger de determinados agravos. A dimensão programática integra os esforços dos programas institucionais, especialmente os programas de saúde. A dimensão social abrange todos os elementos contextuais, relacionados à vida em sociedade: a estrutura jurídica, política e ideológica, as diretrizes governamentais relacionadas à saúde e questões sociais, as relações sociais, as crenças religiosas, entre outros ^14^ .

Após a identificação dos artigos nas bases de dados, foram excluídas as duplicações, seguindo-se a análise por título; por resumo; e do texto na íntegra ( Figura ).

**Figura:** Diagrama de fluxo sobre o processo de seleção dos estudos para revisões de escopo, inspirado em PRISMA por Moher et al. 15 .

### Estágio IV: Mapeamento dos estudos.

Elaboramos um formulário para a extração e sistematização dos seguintes dados: autor, ano da publicação, país do estudo, país de origem e destino dos refugiados, objetivos, tipo e população do estudo, tamanho da amostra, cenário do estudo, elementos de vulnerabilidade nas dimensões individual, programática e social, conforme indicadores do estágio V (Tabelas 2, 3 e 4).

### Estágio V: Conferência, resumo e relatos dos resultados.

Os dados foram analisados e sintetizados segundo os seguintes indicadores de vulnerabilidade, propostos por Nichiata, Takahashi e Bertolozzi ^16^: 

Dimensão individual: 1) Perfil demográfico (sexo, idade, ocupação); 2) Estrutura familiar (nuclear, não nucleada, número de filhos); 3) Condições de vida (domicílio: tendas/containers/casa urbana, saneamento básico); 4) Trabalho (profissão, ocupação, horas de trabalho); 5) Relações sociais no trabalho (proprietário, empregado); 6) Perfil de Morbimortalidade; 7) Crenças e valores sobre o processo saúde-doença.

Dimensão programática: 1) Políticas de Saúde; 2) Ações dos Programas de Saúde; 3) Acesso aos Serviços de Saúde.

Dimensão social: 1) Acesso à: educação, cultura, informação, lazer e justiça; 2) Participação em ações coletivas; 3) Crença religiosa; 4) Preconceito/estigma; 4) Migração (origem, trânsito, destino e retorno).

## RESULTADOS

Da seleção restaram para análise e extração de dados 40 estudos ( Figura ). No processo de análise da produção dos estudos por continente verificou-se que a Ásia produziu 15 estudos (37,5%), a América do Norte 12 (30%), a Europa 12 (30%) e a Oceania 1 (2,5%). Não foi encontrado estudo original publicado em periódico brasileiro ou da América Latina. Também não foi identificada literatura cinzenta relevante. Estudos sobre os sírios foram prevalentes (18: 45%), seguidos dos palestinos (13: 32%) e iraquianos (2: 5%). A predominância de estudos com sírios se justifica pelo intervalo histórico estudado (a partir de 2011). Não identificamos estudos com libaneses refugiados. A maior parte dos estudos (30) foi conduzida com refugiados nos países de destino: Jordânia (17 estudos: 42%); Líbano (9: 22%); Síria (3: 7,5%); em áreas urbanas (29: 72%). A maioria dos estudos (31: 77%) eram quantitativos.

*Vulnerabilidades em saúde dos Refugiados: Categorização por dimensões (individual, programática e social) e país de origem*

### I – Refugiados Sírios

Foram encontrados 22 estudos ( Tabela 2 ) ^17^ . Em relação aos elementos de vulnerabilidade individual, as morbidades que mais se destacaram foram: agravos mentais, sobrepeso/obesidade, desordens alimentares, tuberculose, doenças crônicas não transmissíveis e infecções sexualmente transmissíveis. Os refugiados sírios são cerca de 10 vezes mais suscetíveis que a população do país de acolhida, em relação aos agravos mentais (52%) ^32^ e o transtorno do estresse pós-traumático (TEPT) esteve presente em quase metade dos casos ^28^ , fortemente associado à exposição à guerra ^32^ e a desordens alimentares ^28^ . A ansiedade e a depressão também apresentaram alta prevalência (40,3% e 47,7%, respectivamente). A maior parte dos refugiados que desenvolveram ansiedade e depressão no país de destino não as apresentavam no país de origem ^36^ , quadros que podem decorrer de experiências adversas no destino (exposição a traumas, perda de familiares, em relação ao status social e bens materiais, privação crônica nas necessidades básicas); na transição (meios inseguros de transporte: risco na travessia marítima, de exploração por contrabandistas), nos campos (superpopulação, incerteza em relação ao futuro e grave falta de recursos), e após o reassentamento (falta de familiaridade com o novo contexto, barreiras linguísticas, pobreza, desemprego, falta de apoio, discriminação) ^36^ .

**Tabela 2:** Vulnerabilidades em saúde dos refugiados sírios.

Autor/ano	Tipo de estudo	Objetivo do estudo	População do estudo	Vulnerabilidades identificadas		

				Individual	Programática	Social
UNHCR/UNICEF ^17^ (2016)	Quali-quantitativo *Mixed method* entrevista semiestruturada, Survey e grupo focal.	Fornecer visão multisetorial da situação dos Refugiados sírios no Líbano.	23.000 refugiados sírios, 4.561 crianças menores de 5 anos.	93%: algum grau de insegurança alimentar (1,8 refeições/dia). Doenças crônicas (43%) e mentais (12%). Crianças: 41% doentes nas duas semanas anteriores ao estudo. Sintomas: febre (31%), tosse (25%) e diarreia (15%). Moradias superlotadas e sem banheiro (27%).		84% das crianças de 15 a 17 anos fora da escola. Causas: custo da educação, trabalho e casamento infantil, razões culturais e custo do transporte. 70%: abaixo da linha de pobreza (U$3.84/dia/pessoa).
Hosten E. et al. ^18^ (2018)	Quantitativo Estudo retrospectivo.	Avaliar a prevalência de Tuberculose (TB) ativa e latente, fatores de risco para TB latente e o desempenho do programa de TB jordaniano.	76 refugiados Sírios com TB (casos índice). 481 contatos.	Alta prevalência de TB ativa e latente entre os contatos de paciente com TB. Nos contatos: prevalência de TB ativa e latente, nas crianças menores de 5 anos é 2 vezes maior que entre os adultos.	Taxa de cura entre os casos índice: 28,9%. Entre mulheres: 7,9%. Baixa adesão ao tratamento: 43,4%.	
Truppa. C. et al. ^19^ (2019)	Quantitativo Transversal Survey.	Determinar o impacto da atenção primária em saúde (APS) em populações vulneráveis. Identificar barreiras à utilização dos serviços de saúde.	656 refugiados sírios no Líbano.	Motivos mais comuns para procura do atendimento: Doenças crônicas não transmissíveis (40,6%): artrite, condições musculoesqueléticas, hipertensão e diabetes; problemas de saúde sexual e reprodutiva (28,6%). Doenças transmissíveis que afetam os filhos (37,8%).	Barreira mais importante para a utilização dos serviços: falta de acesso à informação sobre a oferta dos serviços (54,2%). Baixa cobertura vacinal. Baixo nível de atendimento pré-natal e planejamento familiar. 80,9% tiveram que pagar quantias adicionais pelos serviços de saúde na APS.	-
Doocy. S; Lyles, E. ^20^ (2017)	Quantitativo Desenho não identificado Survey.	Identificar necessidades não atendidas e prioridades de assistência em famílias submetidas a deslocamento e chefiadas por mulheres.	2.045 famílias refugiadas Sírias.	Famílias submetidas a deslocamento e chefiadas por mulheres: mais vulneráveis. Insegurança alimentar.		
Ay, M. et al. ^21^ (2016)	Quantitativo Transversal Analítico Observacional.	Identificar as necessidades dos serviços de cuidado à saúde, acessibilidade aos serviços e barreiras no acesso	196 refugiados sírios na Jordânia.	56,6% procuraram a APS por doenças agudas em adultos e 53% em crianças. 36% reportaram doenças crônicas.	Discriminação por parte da equipe. Barreiras no acesso: custo, longas horas de espera, distância ao serviço de saúde, agendamentos tardios para consultas.	Falta de informação sobre a oferta de serviços gratuitos de planejamento familiar.
Al-Rousan, T. et al. ^22^ (2018)	Quali-quantitativo. *Mixed Methods* .	Determinar as necessidades em saúde na perspectiva dos refugiados, equipe de saúde e outros grupos de interesse.	185 refugiados sírios na Jordânia. 75 no campo (41 homens) 110 urbanos (65 mulheres)	Doenças respiratórias associadas ao clima desértico do campo. Doenças crônicas.	Falta de transporte para chegar à UBS. Falta de profissional médico do sexo feminino para o cuidado perinatal. Equipe de saúde sobrecarregada.	Devido ao estigma, não reportam necessidades de cuidados em saúde mental Péssimas condições de moradia.
Cherri, Z. et al. ^23^ (2017)	Qualitativo Grupo focal Entrevista análise temática.	Entender as necessidades, preferências, comportamentos e barreiras à saúde reprodutiva e sexual.	108 mulheres refugiadas sírias (15 a 49 anos) no Líbano.	Alta taxa de natalidade que agrava condição socioeconômica. Casamento precoce (14 anos) devido à escassez econômica.	Custo reportado como barreira ao acesso. Falta de acesso à informação sobre métodos contraceptivos.	42% não utilizavam nenhum método contraceptivo. Migração forçada reportada como causa para não uso.
Doocy, S. et al. ^24^ (2015)	Quantitativo Estudo Transversal.	Caracterizar a prevalência de Doenças Não Transmissíveis.	9.580 refugiados sírios na Jordânia (1.550 famílias) – 0 a mais de 60 anos	50% reportam um membro da família com doença crônica não transmissível.	Políticas de saúde que revogam direitos (em 2014: estabelecido pagamento de taxas para obter cuidado na saúde pública).	Baixo acesso à educação dos chefes de família. Falta de acesso à informação quanto à oferta de serviços de saúde.
Gammoh, O.S. ^25^ (2016)	Quantitativo Estudo transversal.	Descrever queixas, diagnósticos e consumo de medicamentos.	375 refugiados sírios na Jordânia, 0 a mais de 70 anos, 213 Mulheres (56,8%). 162 Homens (43,2%).	Doenças infecciosas, crônicas, cardiovasculares (hipertensão), musculoesqueléticas, gastrointestinais, respiratórias e de pele. Queixas: dor (garganta, gastrointestinal), tosse, falta de ar, rash cutâneo, febre, tonturas, fraqueza, sintomas de ansiedade, problemas dentais e de visão.		
Kakalou, E. et al. ^26^ (2018)	Quantitativo Estudo transversal descritivo.	Descrever o perfil de morbidade da população refugiada na Europa, em 2015–2016.	3.076 sírios, 1.270 iraquianos.	Doenças infecciosas. Condições musculoesqueléticas, de pele, traumas/feridas (causados por acidente/violência). Doenças cardiovasculares, endócrinas, respiratórias, neurológicas, autoimunes e congênitas. Condições dentárias ou da cavidade oral. Condições reprodutivas da mulher, de saúde mental, desnutrição, fraqueza, anemia, escabiose e piolho, abuso de substâncias (psicotrópicos e álcool).	50% das consultas com especialistas foram ofertadas por ONGs (falha na oferta de especialistas pelo sistema de saúde local).	
Sethi. S. et al. ^27^ (2017)	Quantitativo Desenho do estudo não identificado.	Identificar lacunas no cuidado em doenças não transmissíveis.	640 sírios no Líbano. 320 adultos acima de 18 anos. 300 crianças menores de 2 anos.	Doenças transmissíveis e não transmissíveis.	Falta de transporte, de tempo para ir à unidade de saúde e custo: reportados como barreira no acesso.	Baixa escolaridade das mães: 40,3% não têm educação formal. 20% com escolaridade primária completa
Aoun, A; Joundi, J; El Gerges, N. ^28^ (2018)	Quantitativo Estudo transversal.	Examinar a prevalência de transtornos da alimentação e associação com variáveis sociodemográficas e clínicas.	450 Sírios no Líbano. 69 Homens e 381 mulheres (18 a 45 anos).	Transtorno do estresse pós-traumático, desordens alimentares, sobrepeso e desemprego.	-	48% sabem ler e escrever apenas de forma elementar.
Collins, D.R.J. ^29^ (2017)	Quali-Quantitativo *Mixed Methods.*	Determinar o perfil de risco de doenças cardiovasculares da população.	2.907 sírios na Jordânia (18 a > 40 anos) 16 membros de equipe da APS.	Doenças cardiovasculares, dislipidemia, diabetes e sedentarismo.	Conhecimento limitado da equipe de saúde em relação aos protocolos para doenças cardiovasculares.	Padrão migratório impactou a adesão ao tratamento medicamentoso.
Dogru, S; Doner, P. ^30^ (2017)	Quantitativo Retrospectivo e descritivo.	Comparar a frequência de novos casos de tuberculose pulmonar e sucesso no tratamento.	33 refugiados sírios com Tuberculose na Turquia.	Tuberculose acomete faixas etárias mais jovens entre os sírios comparados à população local.	Metas do tratamento para TB não foram alcançadas. Taxa de cura menor nos sírios.	
Doocy. S. et al. ^31^ (2016)	Quantitativo Transversal.	Avaliar o estado de saúde, necessidades não atendidas e a prestação dos serviços de saúde.	1.376 famílias/8.257 Sírios no Líbano (0 a > 60 anos).	Hipertensão, doenças cardiovasculares, diabetes, doenças respiratórias crônicas e artrite.	Necessidade de pagar por serviços de saúde na APS.	
Segal, S. P. et al. ^32^ (2018)	Quantitativo Transversal Entrevista estruturada Survey.	Avaliar o status de saúde mental de refugiados Sírios e Palestinos. Determinar a prevalência de doenças mentais.	161 Palestinos, 47 Sírios e 46 Libaneses no campo de Shatila no Líbano.	Alta prevalência (52%) de doenças mentais (10 vezes maior que a população local).	Necessidade de fortalecimento do programa de saúde mental na APS.	Alto índice de pessoas sem escolaridade: até 3 vezes mais que a população local. Dificuldades no acesso à justiça, leis que restringem os direitos fundamentais. Baixo acesso à economia local. Vivem 17 anos em média no campo. Falta de saneamento básico. Superlotação dos campos. Moradias insuficientes.
Heenan, R.C. et al. ^33^ (2019)	Quantitativo Estudo retrospectivo.	Examinar as condições de saúde de crianças refugiadas sírias e iraquianas no contexto da triagem de chegada na Austrália.	128 crianças (7 meses a 16 anos).	Déficit de vitamina D (63%), déficit no crescimento/nutrição, doenças neurológicas/metabólicas, relativas ao aprendizado/comportamento saúde mental, tuberculose latente, atraso no desenvolvimento,	Os testes de triagem mais omitidos foram para tuberculose (Apenas 7,1% concluíram a triagem). Atraso no calendário vacinal de crianças. Dificuldade no acesso ao teste tuberculínico/PPD na APS.	25% das crianças com dificuldade no acesso à educação mesmo após 3 meses no país de acolhida. Adversidades pré-chegada: exposição a traumas, preocupação com a saúde mental dos pais, preocupação com outras famílias no exterior, separação de um membro da família
Doocy, S. et al. ^34^ (2017)	Quali-Quantitativo *Mixed Method* Estudo Coorte Longitudinal.	Avaliar a eficácia das diretrizes de tratamento e um aplicativo de saúde móvel (mHealth) na qualidade dos cuidados e resultados de saúde	793 Refugiados sírios no Líbano com Diabetes tipo 2 e Hipertensão.	Diabetes descontrolada (57,9%).	Equipe com dificuldade de interação com paciente. Baixo percentual de pacientes que receberam aconselhamento em saúde quanto ao estilo de vida. Divergências entre dados eletrônicos e prontuários escritos.	
Elliot. J.A. et al. ^35^ (2018)	Quantitativo Estudo Transversal.	Determinar associações entre fatores do paciente, autogerenciamento, educação e apoio ao diabetes.	292 Refugiados Sírios no Líbano (> 18 a 84 anos) com Diabetes.	Diabetes (diagnosticada em 30% dos pacientes após o conflito na Síria). Jejum longo (Ramadã) associado ao uso de insulina.		Baixo nível educacional. Incapacidade de reconhecer e responder à hipoglicemia (34%).
Javanbakht. A. et al. ^36^ (2018)	Quantitativo Estudo Transversal.	Determinar a prevalência de desordens mentais.	157 Sírios nos Estados Unidos (18 a 65 anos).	Transtorno do estresse pós-traumático (TEPT) (32,2%), ansiedade (40,3%) e depressão (47,7%), principalmente após a migração forçada.		
Torun. P. et al. ^37^ (2018)	Quali-Quantitativo *Mixed Method.*	Avaliar as necessidades de saúde de refugiados urbanos em Istambul.	111 famílias/8.901 refugiados sírios na Turquia.	Sintomatologia compatível com infecção sexualmente transmissível, doenças crônicas, pulmonares, cardíacas, gastrointestinais, neurológicas, renais, psiquiátricas, musculoesqueléticas, hipertensão.	Gestantes (60%) com dificuldades no acesso devido à barreira linguística, tempo de espera, filas, ambientes tensos e atitudes negativas da equipe.	Alto índice de crianças que abandonaram a escola devido à migração (55%). Falta de ajuda para educação e aluguel. 49,6% não conheciam o direito ao acesso gratuito aos cuidados de saúde. Condições inadequadas de trabalho.
Lyles E. et al. ^38^ (2018)	Quantitativo Desenho não identificado Survey.	Caracterizar o acesso e utilização dos serviços de saúde no Líbano.	2.062 famílias de refugiados sírios no Líbano.	Doenças transmissíveis, e crônicas não transmissíveis, ferimentos, doenças de pele, complicações na gravidez, problemas ginecológicos, gastrointestinais e oftalmológicos.	Maior dificuldade no acesso a medicamentos que a população local.	

Doenças crônicas não transmissíveis, principalmente a diabetes (DM) e hipertensão (HAS) foram mencionadas em oito estudos ^22 , 24 , 27 , 31 , 34 , 35 , 38 , 54^ , sendo que a prevalência de DM foi maior que na população local; nas mulheres, foi quase duas vezes maior, assim como o risco para a doença ^54^ . A maior prevalência de DM refere-se, principalmente, aos indivíduos com história de migração relativamente longa. Verifica-se aumento do risco para DM cerca de seis meses após a chegada, acentuando-se após quatro anos de migração ^54^ . Os refugiados podem enfrentar um acúmulo de fatores de risco para o DM: maior suscetibilidade genética, baixo peso ao nascer, exposição à fome na infância, mudança socioeconômica importante, estresse de aculturação e estilos de vida distintos no país anfitrião. Os requerentes de refúgio com TEPT tiveram chance quase 1,5 vez maior de diagnóstico de DM em comparação a outros na mesma condição ^54^ . Aponta-se, ainda, glicemia acima de 120 mg/dl em jejum em 57,9% dos refugiados com DM ^34^ .

Aproximadamente 73,9% dos refugiados tinham um membro da família com doença crônica (HAS: 25,2% dos pacientes) ^37^ . As doenças respiratórias foram mencionadas em quatro estudos ^22 , 25 , 27 , 31^ . No Líbano, 89,1% dos refugiados sírios da amostra do estudo procuraram a APS devido a doença respiratória crônica ^31^ . Refugiados sofrem exacerbação das doenças respiratórias, devido ao clima desértico de alguns campos de refúgio ^22^ . A tuberculose (TB) foi apontada em três estudos ^27 , 30 , 33^ . Cerca de 11,8% das crianças (com idade entre 7 meses a 16 anos) sírias na Austrália apresentaram tuberculose ^33^ . O sucesso no tratamento da tuberculose foi menor entre os refugiados Sírios na Turquia (63,6% dos casos) do que na população local (88,8%). O contexto de guerra está diretamente relacionado ao aumento da prevalência da enfermidade. Na Síria, antes do início dos conflitos, a incidência era de 23 casos/100 mil habitantes e, após o conflito, aumentou para 51 casos/100 mil ^30^ . Durante a guerra civil, os principais problemas em relação à tuberculose referem-se ao diagnóstico, tratamento e prevenção da doença, ao acesso aos serviços de saúde e ao fornecimento de medicamentos, aumento da reativação, transmissão, devido às condições de vida: aglomeração, nutrição, escassez de medicamentos e de pessoal de saúde, e estresse psicológico ^30^ . As doenças infecciosas/transmissíveis foram mencionadas em três estudos ^25 , 26 , 38^ , sendo que, em estudo realizado no Líbano, com 1.218 refugiados sírios, essas enfermidades foram prevalentes (40,5%) ^38^ .

O indicador de vulnerabilidade “trabalho” foi verificado em três estudos ^27 , 28 , 37^ . A taxa de desemprego entre os refugiados sírios foi de 84,2% na população do estudo ^28^ . O indicador “crenças e valores” sobre a doença foi verificado em quatro estudos ^23 , 28 , 29 , 35^ . Apenas 58% das mulheres sírias no Líbano usavam algum método anticonceptivo; a taxa de natalidade é alta, destacando-se que mulheres acima de 40 anos reportaram ter entre oito e 12 filhos ^23^ .

O indicador “condições de vida” foi constatado em cinco estudos ^17 , 20 , 22 , 32 , 56^ , verificando-se insegurança, moradias superlotadas e ausência de banheiros em campo de refúgio ^17 , 22^ . Mais da metade, 54,3%, relatou uso de mecanismos extremos de enfrentamento da insegurança alimentar (redução da porção; do número de refeições consumidas/dia; consumo reduzido pelos adultos para permitir a alimentação das crianças) ^20^ .

Em relação à dimensão programática, o indicador “acesso aos serviços de saúde” foi o mais constatado nos estudos (8) ^21^ , seguido do indicador “políticas de saúde” (7) ^22^ e do indicador “ações dos programas” (6) ^21 , 29^ . Em relação ao acesso aos serviços de saúde, 60% das gestantes sírias na Turquia enfrentaram dificuldades, devido ao idioma e ao tempo de espera para atendimento ^37^ . A falta de profissionais médicas foi reportada como barreira ao acesso para o cuidado perinatal ^22^ e também o custo ^21 , 23 , 24 , 38^ . Na Grécia, um estudo apontou que 50% das consultas em especialidades médicas foram realizadas por ONGs nacionais e internacionais, evidenciando transferência de responsabilidade, do governo local para essas entidades, em providenciar acesso aos serviços de saúde. As principais causas de encaminhamento para serviços de referência são as obstétricas/ginecológicas (35%), cuidados pediátricos (15%), e para obter medicamentos prescritos não disponíveis na APS (16%) ^26^ .

Em relação ao indicador “Políticas de Saúde”, destaca-se a falta de um sistema universal e gratuito ^22 , 27 , 56^ . Em estudo com refugiados sírios na Jordânia, 80% dos entrevistados relataram a necessidade de pagar alguma quantia para cuidados em saúde na APS ^22^ .

Quanto ao indicador “ações dos programas de saúde”, constantes da dimensão programática, evidencia-se a dificuldade das equipes de APS na interação com os refugiados sírios ^34^ , principalmente no Líbano. Cerca de 40% dos entrevistados na Jordânia reportaram discriminação por parte da equipe de saúde ^21^ . Deve-se ponderar que profissionais de saúde sofrem a tensão decorrente de processos estruturais e da frágil governabilidade local, expondo-os aos extremos da escassez de recursos humanos e materiais e, principalmente de conhecimento sobre o tema do refúgio na saúde ^56^ .

Na dimensão social constatou-se o indicador “acesso à educação” ^21 , 23 , 24 , 27 , 28 , 32 , 33 , 35 , 37 , 56^ , sendo que quatro desses estudos ^21 , 24 , 28 , 37^ tratam especificamente da dificuldade de acesso aos estudos por parte da população adulta refugiada. Em um dos estudos, 42% não completaram nenhuma educação formal ^35^ . Detectou-se ainda um alto índice (55%) de abandono escolar pelas crianças ^21^ . Na Austrália, um estudo reportou que 25% das crianças sírias encontravam-se fora da escola, mesmo após três meses no país e 67,9% perderam pelo menos um ano de atividades escolares antes de chegar no país ^33^ .

O indicador “acesso à informação” também foi verificado ^21 , 23 , 24 , 35 , 37^ , identificando-se falta de informação quanto à disponibilidade dos serviços de planejamento familiar na UBS ^21 , 23^ . Em um dos estudos com refugiados sírios no Líbano, apontou-se que 61% dos entrevistados não sabiam da disponibilidade dos serviços ^21^ . Por outro lado, ONGs internacionais e locais providenciam cuidados para mulheres sírias por meio da APS, oferecendo planejamento familiar sem custos (inserção de DIU, pílulas contraceptivas e preservativos masculinos), além de consulta com parteira ou obstetra/ginecologista e testes laboratoriais ^21^ .

O marcador “migração” foi constatado em três estudos ^23 , 37 , 56^ . O padrão do fluxo migratório impacta a adesão ao tratamento de doenças cardiovasculares em refugiados sírios que chegam à Jordânia, pois durante a viagem não conseguem adquirir os medicamentos ^29^ . Nesse país, um estudo apontou que 50% dos sírios tinham planos concretos para deixá-lo, principalmente em direção à Europa, em decorrência da falta de acesso a direitos fundamentais, entre os quais educação, saúde, trabalho e alimentação. Destaca-se parte de um depoimento: “ *uma rápida morte no mar, é melhor do que morrer mil mortes todos os dias* ” ^56^ .

### II – Refugiados Palestinos

A Tabela 3 sumariza os 14 estudos que tratam dessa população refugiada ^39^ . O diabetes foi a enfermidade que mais se destacou ^40 , 42 , 43 , 45 , 48 , 50 , 52^ . Em um estudo com 2.851 palestinos com idade até 60 anos e alocados na Jordânia, a associação entre o DM e a HAS chega a 77% da amostra ^50^ e a obesidade é um dos principais fatores adicionais de risco associado. Em outro estudo, dos pacientes diabéticos 59% eram obesos (IMC > 30), sendo 69% mulheres ^52^ . Uma das pesquisas com refugiados palestinos na Jordânia, mostrou a associação entre DM e HAS em 81% da amostra, acometendo todas as faixas etárias: 21% eram menores de 5 anos, 36% entre cinco e 10 anos e 43% eram maiores de 10 anos de idade ^42^ . A hipertensão também se destaca entre as principais morbidades ^42 , 44 , 45 , 48 , 50 , 52^ , com aumento da prevalência, de 2008 a 2012, entre refugiados palestinos na Jordânia (dois milhões), variando de 13,8% a 16,3% dos pacientes atendidos. Destacam-se pacientes com hipertensão sem controle que, no mesmo período, variou entre 31,5% e 45,9% dos pacientes ^44^ .

**Tabela 3:** Vulnerabilidades em saúde dos refugiados Palestinos.

Autor/ano	Tipo de estudo	Objetivo do estudo	População do estudo	Vulnerabilidades identificadas		

				Individual	Programática	Social
Al Baz, M. Law, M. R. Saadeh, R. ^39^ (2018)	Quantitativo Estudo Transversal Survey.	Estudar o conhecimento, atitudes e comportamentos sobre o uso de antibióticos.	250 palestinos na Jordânia. Média: 32 anos 205 mulheres e 45 homens acima de 18 anos.	47% uso de automedicação com antibióticos; Desemprego; Renda abaixo da média nacional.	Horas de espera no serviço de saúde.	-
Khader. A. et al. ^40^ (2014)	Quantitativo Coorte.	Relatar as complicações de diabetes de uma coorte de refugiados.	119 palestinos diabéticos na Jordânia de 0 a > 60 anos.	Diabetes, obesidade, sedentarismo, doenças cardiovasculares. 57% tinham risco adicional de obesidade (IMC > 30).	Perda progressiva no acompanhamento do tratamento (p < 0,001) Redução progressiva na proporção de pacientes que frequentavam a clínica a cada ano (p < 0,001)	-
Riccardo, Flavia; Khader, Ali; Sabatinelli, Guido ^41^ (2011)	Quantitativo Estudo Transversal Survey.	Analisar as tendências da mortalidade infantil, entre refugiados Palestinos (1995–2005).	14.202 crianças na Jordânia, Líbano, Síria, Faixa de Gaza e Westbank.	Taxa de mortalidade infantil maior que a da população local. Baixo Peso/ Prematuridade. Malformação congênita associada à gravidez precoce e casamento consanguíneo. Infecções respiratórias.	Acesso precário à saúde: principal condicionante para morte neonatal. Cuidado fragmentado, sistema de saúde altamente privatizado.	-
Khader. A. et al. ^42^ (2014)	Quantitativo Coorte.	Determinar os resultados do tratamento de diabetes mellitus (DM).	2.246 palestinos na Jordânia de 0 a > 60 anos com DM.	81%: Diabetes + HAS. 58%: DM controlada	Dificuldade da equipe em manter o acompanhamento do tratamento.	
Al Kasseh, A. S. M. et al. ^43^ (2013)	Quantitativo Caso controle retrospectivo.	Determinar os fatores de risco para diabetes gestacional em mulheres refugiadas.	189 palestinas na Faixa de Gaza. 18 a 36 anos/Idade Média 34.1 anos.	Fatores de risco ao Diabetes gestacional: histórico de abortamento espontâneo (mais que 1) (p < 0,0001); Peso antes da gravidez (p < 0,001); Histórico familiar de DM (p < 0,001); Histórico de natimorto (p = 0,002); Peso na gravidez (p < 0,001).	Fragilidade de políticas preventivas no período pós-parto.	Baixo nível educacional foi fator associado (p < 0,001) a Diabetes gestacional.
Saadeh. R. et al. ^44^ (2015)	Quantitativo Desenho do estudo não especificado.	Descrever as tendências de utilização de anti-hipertensivos.	244.169 palestinos na Jordânia > 40 anos.	Aumento da prevalência da hipertensão de 2008 a 2012.	Aumento dos gastos do programa com anti-hipertensivos.	
Khader. A. et al. ^45^ (2014)	Quantitativo Coorte.	Determinar características dos refugiados e do programa de Hipertensão.	18.881 palestinos na Jordânia > 60 anos, com Hipertensão	Hipertensão, diabetes, obesidade, cardiopatia congestiva, sedentarismo, infarto agudo do miocárdio, acidente vascular cerebral e cegueira.		
Abouteir. A. et al. ^46^ (2011)	Quantitativo Caso controle prospectivo.	Investigar a relação entre a freqüência de diarréia e fatores de risco potenciais, incluindo o acesso à água.	3.338 palestinos na Faixa de Gaza. 266 pacientes com diarreia, de 0 a > 40 anos.	Infecção respiratória e diarreia. Fatores associados à diarreia (p < 0,05): Status socioeconômico, pobreza, desemprego, falta de acesso a água pública, animais domésticos, saneamento básico precário.	Terapia medicamentosa prescrita não segue as recomendações da OMS.	
Alameddine. M. et al. ^47^ (2019)	Qualitativo Entrevista semiestruturada análise temática.	Compreender a resiliência dos sistemas de saúde.	61 palestinos no Líbano e Jordânia; 37 em campo de refúgio/ 24 fora do campo;	TEPT, violência contra mulher, depressão.	Equipe de saúde sobrecarregada e alto índice de absenteísmo. Estresse no ambiente de trabalho.	
Khader. A. et al. ^48^ (2012)	Quantitativo Coorte.	Registrar, monitorar e relatar doenças crônicas.	4130 palestinos na Jordânia com Hipertensão de 0 a > 60 anos.	Diabetes, doenças cardiovasculares e hipertensão.	Falha na medida de pressão arterial e testes de glicemia.	
Bastin. P. et al. ^49^ (2013)	Quantitativo Coorte.	Identificar agravos mentais.	1.107 palestinos, iraquianos e libaneses – 18 a > 60 anos.	28%: depressão 15,6%: ansiedade 11,5%: Psicoses.		Falta de acesso à educação associada a problemas de saúde mental.
Khader. A. et al. ^50^ (2012)	Quantitativo Coorte.	Avaliar a qualidade dos serviços para refugiados com diabetes.	2.851 palestinos na Jordânia, com Diabetes de 0 a > 60 anos.	Diabetes, cegueira, acidente vascular cerebral, doenças cardiovasculares e amputações.	Falha em manter adesão ao tratamento após período de 12 meses. Falha da equipe de saúde no seguimento de protocolos.	
Saleh. S. et al. ^51^ (2018)	Quantitativo Estudo Clínico Randomizado Controlado.	Avaliar o efeito de ferramentas de saúde móvel de baixo custo na acessibilidade aos serviços de saúde.	2.359 palestinos no Líbano 1.433 intervenção 926 controles 40 a > 71 anos com DM e HAS.	Diabetes e hipertensão.	O programa demonstrou fragilidades: manter o checkup oftalmológico anual em pacientes com diabetes e hipertensão e na promoção da cessação do tabagismo. Os SMSs não geraram um efeito de intervenção claro na utilização dos serviços de APS.	
Khader. A. et al. ^52^ (2013)	Quantitativo Coorte.	Relatar o número e as características de novos refugiados com DM.	12. 550 palestinos na Jordânia com Diabetes de 0 a > de 60 anos.	Diabetes tipo I e II, hipertensão, tabagismo, sedentarismo, obesidade. Complicações do diabetes: infarto agudo do miocárdio, doença renal em estágio final.		

Em relação aos agravos mentais, destaca-se a depressão (28%) ^47 , 49^ e dentre as doenças infecciosas, chamam atenção as diarreias. Um dos estudos aponta que 20,3% estavam infectados com *Giardia duodenalis* , relacionada à baixa qualidade da água. A falta de acesso à água potável pública e gratuita foi associada às diarreias infecciosas, destacando-se que 62,4% tiveram que comprar água ^46^ .

Um dos estudos, realizado na Jordânia, Líbano, Síria, Gaza e Cisjordânia, identificou as principais causas de morte infantil no primeiro ano de vida: baixo peso e prematuridade (30%), malformação congênita (22%) e infecção do trato respiratório (13,4%) ^41^ .

O indicador “trabalho” foi identificado em dois estudos ^39 , 46^ . A taxa de desemprego chega a 90% entre os refugiados palestinos na Jordânia ^39^ . Mesmo no próprio território palestino, a maioria daqueles que conseguiram emprego, tinha relações informais de trabalho ^46^ .

O indicador “condições de vida” foi verificado em dois estudos ^32 , 46^ . Os palestinos vivem no campo de refúgio em média 16,2 anos, chegando até a 27,5 anos, período muito maior do que os sírios. Nesse contexto, vivem sem serviços públicos de saneamento, as casas/tendas/containers são insuficientes, além de verificar-se superlotação ^32^ .

Sobre a vulnerabilidade programática, foi preponderante o indicador “ações dos programas de saúde” ^40 , 41^ . Destaca-se o relato de equipes e pacientes que apresentam sobrecarga mental e física no trabalho ^47^ . É relevante a menção sobre a perda do acompanhamento no tratamento de DM e HAS ^40 , 42 , 48 , 50^ . O aumento do fluxo migratório tem reflexo direto na oferta dos serviços. Na Jordânia, dos pacientes que recorreram à APS, 58% não conseguiram ter a glicemia pós-prandial mensurada ^50^ . Entre os pacientes com hipertensão, 37% não tiveram a pressão arterial registrada ^50^ e 38% faltaram ao retorno programado ^40^ .

O segundo indicador mais constatado refere-se às “políticas de saúde” ^43 , 44 , 47^ , voltadas principalmente à intervenção medicamentosa, com repercussões nos gastos com hipertensão ^44^ . Apenas em 2011 a ONU introduziu equipes de saúde da família na Síria, Líbano e Jordânia; antes, as intervenções limitavam-se à centralidade médica ^47^ .

Dois estudos trataram do indicador de vulnerabilidade programática “acesso aos serviços de saúde” ^39 , 41^ . Em um deles, a maioria, 88%, dos refugiados palestinos na Jordânia relatou espera de longas horas na unidade de saúde, o que resultou na duplicação do risco de automedicação com antibióticos ^39^ . Já na Faixa de Gaza e Cisjordânia, o acesso precário aos serviços de APS foi o principal condicionante de morte neonatal ^41^ .

Em relação aos elementos de vulnerabilidade social, o indicador mais verificado foi “acesso à educação” ^32 , 43 , 49^ . Um dos estudos aponta que os refugiados palestinos sem nenhum grau de escolaridade no Líbano foram 15,5% da amostra, quase o dobro da população libanesa local com o mesmo grau de escolaridade (8%) ^32^ . O baixo nível educacional entre as refugiadas palestinas grávidas na Faixa de Gaza foi fator associado à predisposição ao diabetes gestacional ^43^ . Os estudos confirmam a exclusão social, em relação à participação na economia, com subempregos de caráter informal, nesse grupo de refugiados. Restrições nas leis, nos países de acolhida, como por exemplo, no Líbano, corroboram o estigma sobre os palestinos.

### III – Refugiados Iraquianos

A Tabela 4 sumariza os cinco estudos referentes a essa população refugiada ^53^ . Destaca-se a prevalência de doenças infecciosas, desnutrição, agravos mentais e diabetes. Nos adultos, a prevalência de DM foi maior que na população local e quase duas vezes maior em mulheres ^54^ . Em crianças, destacam-se o déficit de vitamina D; problemas relativos ao crescimento e nutricionais; tuberculose latente; problemas neurológicos/metabólicos e de aprendizado/comportamento; atraso no desenvolvimento; além da exposição a traumas e separação de um membro da família ^33 , 53 , 54^ .

**Tabela 4:** Vulnerabilidades em saúde dos refugiados Iraquianos.

Autor/ano	Tipo de estudo	Objetivo do estudo	População do estudo	Vulnerabilidades identificadas		

				Individual	Programática	Social
Worabo, H.J. et al. ^53^ (2016)	Qualitativo Grupo focal em profundidade. Análise Fenomenológica.	Entender a percepção dos refugiados recém-chegados a respeito do sistema de saúde americano.	10 iraquianos (> 18 anos) nos EUA; 7 Homens.	Doenças infecciosas, desnutrição e problemas mentais. Relutância em aderir ao tratamento da tuberculose latente por não apresentarem sintomas e não se sentirem doentes.	Barreiras linguísticas causaram abandono do tratamento. Falta de intérprete tradutor. Equipe não escuta as queixas. Longa espera para consulta na APS.	Não procuram a APS, pois não podem pagar tradutor e devido ao alto custo dos serviços de saúde.
Goosen. S. et al. ^54^ (2014)	Quantitativo Estudo populacional descritivo.	Mapear a prevalência e incidência de diabetes.	9.436 Iraquianos na Holanda. 1.169 Sírios. Faixa etária: 20 a 79 anos.	Diabetes: duas vezes mais prevalente entre refugiados comparados à população local		
Van Loenen. T. et al. ^55^ (2018)	Qualitativo Entrevista Semi estruturada/Análise temática.	Compreender as necessidades em saúde, barreiras no acesso e desejos dos refugiados em relação à APS.	39 sírios, 12 Iraquianos na Grécia, Eslovênia, Croácia, Hungria, Holanda, Itália, Áustria.		Falta de oferta de serviços de saúde mental na APS. Falta de continuidade no cuidado. Dificuldades em encontrar atendimento médico nas fronteiras que apresentam maior movimento.	
Kvittingen. A. et al. ^56^ (2018)	Qualitativo Entrevista em profundidade.	Entender as experiências de refugiados sírios e iraquianos e suas aspirações migratórias.	62 refugiados na Jordânia: 32 Iraquianos e 30 Sírios.	Falta de emprego formal. Nenhum dos entrevistados conseguiu residência legal.	Políticas de revogação ao livre acesso à saúde e educação públicas e limitação ao acesso à assistência humanitária internacional.	50,0% dos entrevistados tinham planos concretos para deixar o país devido a restrições e falta de oportunidades.
Doocy.S; Burnham G. ^57^ (2011)	Quantitativo Desenho não identificado.	Oferecer informação sobre a economia familiar e meios de subsistência de refugiados iraquianos em área urbana da Síria.	800 famílias de iraquianos na Síria.	Superlotação das acomodações. Apenas 12% com trabalho estável; trabalho pesado; trabalho infantil; elevada jornada de trabalho/semana; subpagamento ou não pagamento pelo trabalho realizado.	Desconhecimento sobre o acesso a serviços de planejamento familiar.	Pobreza relacionada ao baixo status educacional.

Outro indicador verificado foi o “trabalho” ^53 , 56 , 57^ . Em um estudo com refugiados iraquianos nos Estados Unidos, o desemprego chegou a 90% ^53^ , com predomínio da informalidade. A maioria (61%) dos refugiados iraquianos na Síria tem trabalho casual, no setor de serviços (comércio) e apenas 12% têm trabalho formal e estável. É expressivo o trabalho árduo e média da jornada de 59 horas semanais. Também se verificou relatos de trabalho infantil, subemprego e pagamento pela mão de obra ofertada muito abaixo do mercado, além da falta de pagamento após prestação de serviços ^56^ .

Em relação ao indicador “crenças e valores”, destaca-se a não adesão ao tratamento da tuberculose latente. A ausência de sintomatologia leva a acreditar que o tratamento é desnecessário ^53^ .

Em relação à dimensão programática da vulnerabilidade, destaca-se o indicador “ações dos programas de saúde” ^33 , 53 , 55^ , principalmente concretizadas por longas horas de espera (nos serviços de saúde americanos), além de falta de tradutor e de empatia por parte da equipe de saúde ^53^ . Em um dos estudos, apenas 1,8% das crianças tiveram avaliação completa da saúde, segundo o protocolo preconizado para os refugiados ^33^ .

Sobre o “acesso aos serviços de saúde” ^33 , 55 , 57^ verificou-se dificuldade para realizar o teste tuberculínico entre os refugiados iraquianos na APS da Austrália; e no acesso ao atendimento médico em fronteiras mais movimentadas da Europa ^55^ . Ademais, muitos pacientes não usavam método contraceptivo e 82% não conseguiam acessá-lo por desconhecer os serviços disponíveis de planejamento familiar ^57^ .

Quanto ao indicador “políticas de saúde”, um estudo menciona que a Jordânia revogou o livre acesso de refugiados iraquianos à saúde e aos diretos fundamentais, um retrocesso nessa oferta gratuita de atendimento em saúde, motivado pelo discurso de déficit financeiro ^56^ .

O indicador mais verificado, em relação à dimensão social da vulnerabilidade, refere-se ao “acesso à educação” ^33 , 49 , 56 , 57^ . Cerca de 32% das crianças iraquianas na Austrália perderam três ou mais anos de escola no período pré-chegada ao país ^33^ . As crianças têm dificuldade na matrícula escolar por falta de endereço residencial oficial na Jordânia ^56^ . Os refugiados com menor escolaridade foram mais suscetíveis às doenças mentais ^49^ , à pobreza, e cerca de duas vezes mais suscetíveis a renda per capita abaixo de US$ 1/dia ^57^ . O indicador “migração” traz à tona o sentimento de ameaça constante, por medo de expulsão pelo governo, experimentado pelos refugiados iraquianos na Jordânia ^56^ .

## DISCUSSÃO

Todas as dimensões da vulnerabilidade se fizeram presentes nos estudos de nossa amostra. Nos achados bibliométricos aponta-se que o predomínio (77%) de estudos quantitativos pode resultar em avaliações parciais sobre as experiências de vida das populações estudadas. Também foram escassos (8%) os estudos realizados integralmente nos campos de refúgio e estudos com amostras significativas no Brasil e América Latina, além da escassez de estudos multicêntricos. Não houve menção nos estudos ou análise comparativa, em relação à diferença no acesso à saúde entre os refugiados e os solicitantes de refúgio. Também não houve estudo sobre análise comparativa das vulnerabilidades em saúde entre refugiados árabes e refugiados de outras nacionalidades. É importante destacar que os “refugiados” e os “solicitantes de asilo” compõem o mesmo bloco de migrações forçadas, mas os solicitantes apenas aguardam uma etapa burocrática para seu reconhecimento como refugiado no país de acolhida.

Entre os sírios constata-se lacuna de estudos sobre as necessidades em saúde de grupos específicos, como crianças, adolescentes e mulheres, não tendo sido identificados estudos sobre as necessidades em saúde de mulheres refugiadas palestinas na APS. Apesar de a Turquia ser uma das principais rotas de destino dos sírios, observou-se em nossa amostra escassez de estudos (5%) relacionados ao contexto da APS nesse país de acolhida.

Em relação às dimensões da vulnerabilidade, a dimensão individual revela a presença de enfermidades crônicas e infecciosas, além da importante presença de agravos mentais, que são corroborados e atribuídos a experiências traumáticas, antes mesmo da migração forçada: violência, abuso e incerteza em relação ao futuro ^58^ . Apesar da expressividade dos agravos mentais, esta revisão e outros estudos apontam um novo desafio referente à escalada das DCNT, com repercussões sobre o sistema de saúde dos países de acolhida nas áreas de conflito, principalmente na Jordânia, Líbano e Turquia, e aumento dos gastos para o tratamento, principalmente da DM e HAS ^22 , 24 , 27 , 31 , 34 , 35 , 38 , 44 , 54 , 59^ . Destaca-se que nos estudos analisados nesta revisão, a APS teve papel central em proporcionar o acesso aos sistemas de saúde dos países vizinhos aos conflitos ^47 , 60^ .

Os achados relativos à saúde das mulheres são corroborados por estudo que identificou diabetes gestacional, natimortos e crianças com baixo peso ao nascer, além de pré-natal inadequado ^61^ . A malformação congênita foi associada à gravidez precoce e ao casamento consanguíneo em refugiadas palestinas ^41^ . Crianças refugiadas sofrem as condições decorrentes de alimentação inadequada, desnutrição, deficiências de micronutrientes, além de necessidades de saúde bucal e doenças infecciosas, em adição às decorrências de exposição a conflitos bélicos, violência e xenofobia, assim como atraso no desenvolvimento e falhas na escolaridade, entre outros ^62^ . Achados recentes apontam evidente desigualdade na cobertura vacinal em crianças refugiadas quando comparadas à população em geral: elas têm três vezes menos chance de serem vacinadas contra infecções evitáveis, principalmente contra o sarampo, tétano e a meningite C ^63^ . Evidencia-se, no presente estudo, a dificuldade no acesso às vacinas e baixa cobertura vacinal já na chegada aos países de acolhida ^33^ .

Em relação à dimensão programática, destaca-se o esfacelamento das políticas, dos programas, dos serviços e das equipes de saúde. As equipes de APS que atuam na linha de frente, em regiões de grandes fluxos migratórios, apresentam dificuldades em lidar com os refugiados e sobrecarga de trabalho, além de absentismo ^47^ . Outro estudo corrobora e aponta esse fenômeno como consequência dos conflitos e da guerra, que impactam todo o sistema de saúde local e dos países vizinhos, com redução na oferta e na qualidade dos serviços, assim como êxodo de profissionais de saúde. Como reflexo, são adotadas políticas de exclusão que restringem e burocratizam o acesso à APS, tais como maior exigência de documentos para a aquisição de auxílios moradia e alimentação, fim da gratuidade e início de cobrança nos serviços de APS ^64^ . Há desigualdade no acesso, principalmente para os refugiados com menor nível educacional, que são mais vulneráveis entre os vulneráveis e que dependem de assistência dos governos locais ^17 , 24 , 27 , 32^ . Estudo transversal com 400 refugiados sírios no Canadá aponta que refugiados que acessam o serviço de saúde público ou financiado por governos locais, quando comparados a refugiados que acessam o sistema de saúde privado ou financiado pela iniciativa privada, reportam mais necessidades em saúde não atendidas e condições médicas mais complexas e são quase três vezes mais suscetíveis (OR = 2,84; IC95% 1.55–5.20) a não terem suas necessidades em saúde atendidas. Desses refugiados, apenas 58% reportam ter um médico de família de referência ^65^ . Há relatos sobre a necessidade crescente de que os refugiados no Líbano e na Jordânia paguem pelos serviços de APS ^22 , 27 , 56^ . Embora os refugiados desembolsem menos que a população local, destaca-se que tal situação soma-se ao alto índice de desemprego, e à baixa renda (menor que dois dólares/dia entre os refugiados) ^22^ . A ONU/ACNUR oferece um programa de distribuição de renda e assistência financeira na Jordânia para os refugiados sírios, por exemplo, mas apenas 23 mil famílias têm acesso ^22^ , quantitativo muito restrito considerando-se mais de 676.300 refugiados sírios sob tutela da ONU naquele país ^4^ . Na Jordânia é necessário um cartão biométrico de identificação pessoal para o acesso à saúde e suporte nutricional, mas há uma série de exigências para a sua obtenção ^17 , 22^ . Em decorrência, muitos sírios foram forçados a reduzir ainda mais o aporte de alimentos, deixar de buscar cuidado em saúde e retirar as crianças da escola para compensar custos ou gerar renda adicional por meio do trabalho infantil ^17^ . A média de refeições de refugiados adultos sírios no Líbano era de 1,8 refeições/dia, e entre as crianças, de 2,3 refeições/dia ^17^ .

Quanto aos principais países de destino e acolhida, a Jordânia, o Líbano e a Síria, não há informações explícitas nas páginas de seus respectivos ministérios da saúde sobre políticas públicas de saúde para refugiados. O Líbano não é signatário da Convenção de Refugiados de 1951 e, nesse sentido, não há lei doméstica que atenda à necessidade dos refugiados no país ^68^ . Há registros de que Palestinos e Sírios sofrem com a marginalização e discriminação como resultado de políticas que negam o acesso aos direitos básicos, como moradia, trabalho, educação e saúde ^69^ . A *United Nations Relief and Works Agency* (UNRWA) tem sofrido cortes orçamentários importantes e seu maior doador, os Estados Unidos, suprimiu o financiamento ^69^ . Entretanto, há controvérsias sobre as ações governamentais em relação aos refugiados nesse país, apontando a existência de provisão de cuidados primários à saúde, que inclui consultas, exames laboratoriais e testes diagnósticos para grupos previamente definidos como vulneráveis, oferecidos por meio de custo reduzido aos residentes ^69^ . A vacinação, dois exames de ultrassom para gestantes e medicação para agravos agudos e crônicos são gratuitos ^69^ .

Na Jordânia, aponta-se que o aluguel de moradias é acessível e a moradia em assentamentos é oferecida em troca de trabalho em fazendas locais ^69^ . A oferta reduzida de trabalho determina a necessidade de trabalhar ou casar-se, o que leva ao abandono da educação ^69^ . Para mitigar tais situações, o governo implementou um programa: “Cash+”, que inclui intervenções de proteção social para as famílias em situação de vulnerabilidade, que recebem, mensalmente, transferência monetária incondicional por criança. Programas voltados à saúde mental também são oferecidos em integração aos serviços de atenção primária à saúde ^69^ . Os refugiados nos campos têm livre acesso à saúde, que é subsidiada pelo governo e por agências internacionais. Refugiados registrados no Ministério do Interior na Jordânia têm acesso a cuidados de saúde e benefícios governamentais da mesma forma que jordanianos sem seguro. Entretanto, salienta-se que os refugiados apresentam sobrecarga financeira para cobrir consultas e medicação em serviços privados de saúde ^70^ .

Quanto à dimensão social da vulnerabilidade, evidenciou-se desigualdade no acesso à educação, à informação, ao trabalho digno, além de preconceito/estigma e dificuldades de participação em ações coletivas e consequente dificuldade de integração nos países de acolhida ^17 , 21 - 24 , 27 , 32 , 33 , 35 , 37 , 43 , 49^ . De fato, a desconexão social e comorbidades são prevalentes e a falta de engajamento na comunidade foi associada a desfechos desfavoráveis em saúde, principalmente em relação à saúde mental. A dificuldade de integração social persistiu por três ou mais décadas após a chegada nos EUA, constituindo-se como fator de risco à saúde ^66^ . A exclusão social dos refugiados é apontada, por outros achados, como consequência de desigualdades estruturais, dentre elas, políticas de marginalização e falta de seguridade social. A escassez de serviços básicos, nos países de acolhida, provoca disputa entre a população local e os refugiados, além de progressiva degradação das condições de vida ^67^ .

No que se refere às recomendações para políticas e práticas na APS, há necessidade de rastreio de casos e fortalecimento dos programas e políticas, especialmente no campo da saúde mental, com refugiados sírios e palestinos ^36^ . Evidências sugerem a indicação de triagem de diabetes para os refugiados recém-chegados com idade maior que 35 anos ^54^ . Os profissionais de saúde devem estar conscientes do alto risco para diabetes entre os refugiados sírios, palestinos e iraquianos ^54^ . Ademais, aponta-se a necessidade de fortalecer os programas de saúde da mulher, visando identificar precocemente o diabetes gestacional, inclusive no período pós-parto, principalmente com refugiadas palestinas ^43^ . Para melhorar a adesão e o sucesso no tratamento da tuberculose entre os refugiados, aponta-se a necessidade de programa específico ao grupo e que aborde principalmente as crenças sobre a doença e o tratamento da tuberculose latente ^30^ .

Uma das limitações desta investigação é a restrição a estudos com sujeitos que migraram forçadamente, portanto, as vulnerabilidades mapeadas não são generalizáveis para o bloco das migrações não forçadas. Ademais, os elementos de vulnerabilidade encontrados não abarcam o contexto dos 22 países árabes do globo, restringindo-se aos sírios, palestinos e iraquianos. Também é importante apontar a heterogeneidade dos métodos utilizados, o que dificultou o processo de extração dos dados, inclusive quando o estudo tratava de refugiados de mais de um país de origem árabe, pois alguns não separaram desfechos por nacionalidade, o que dificultou a extração e a síntese da presente revisão.

## CONCLUSÃO

O continente asiático, o Oriente Médio, seguido pela América do Norte e União Europeia foram os que mais produziram estudos com refugiados árabes no contexto da APS. Há uma evidente lacuna na produção do conhecimento a respeito, no continente latino-americano e nenhum dos estudos utilizou o conceito de vulnerabilidade, conforme foi adotado na presente revisão de escopo.

Os refugiados árabes vivenciam contextos de alta vulnerabilidade, colocando-os em profunda desigualdade e desvantagem perante os programas, serviços e sistema de saúde do país de acolhida. Portanto, destaca-se a necessidade de programas e políticas que considerem os elementos de vulnerabilidade e promovam ações na APS, de forma a responder às necessidades de saúde dos refugiados. É necessário considerar que o embate de forças entre os países, fundamentalmente por interesses econômicos, causa repercussões destrutivas para as populações, impondo sua urgente superação e intransigência, além do repúdio a ações que evidenciem injustiça social. Defende-se a necessidade de políticas para a inclusão, justiça social e condições dignas de vida para a totalidade das pessoas em situação de refúgio, rechaçando-se todo tipo de atitude e práticas estigmatizantes e que evidenciem desumanização.
